# *ttm-1* Encodes CDF Transporters That Excrete Zinc from Intestinal Cells of *C. elegans* and Act in a Parallel Negative Feedback Circuit That Promotes Homeostasis

**DOI:** 10.1371/journal.pgen.1003522

**Published:** 2013-05-23

**Authors:** Hyun Cheol Roh, Sara Collier, Krupa Deshmukh, James Guthrie, J. David Robertson, Kerry Kornfeld

**Affiliations:** 1 Department of Developmental Biology, Washington University School of Medicine, St. Louis, Missouri, United States of America; 2 Research Reactor Center, University of Missouri, Columbia, Missouri, United States of America; 3 Department of Chemistry, University of Missouri, Columbia, Missouri, United States of America; University of Wisconsin, United States of America

## Abstract

Zinc is an essential metal involved in a wide range of biological processes, and aberrant zinc metabolism is implicated in human diseases. The gastrointestinal tract of animals is a critical site of zinc metabolism that is responsible for dietary zinc uptake and distribution to the body. However, the role of the gastrointestinal tract in zinc excretion remains unclear. Zinc transporters are key regulators of zinc metabolism that mediate the movement of zinc ions across membranes. Here, we identified a comprehensive list of 14 predicted Cation Diffusion Facilitator (CDF) family zinc transporters in *Caenorhabditis elegans* and demonstrated that zinc is excreted from intestinal cells by one of these CDF proteins, TTM-1B. The *ttm-1* locus encodes two transcripts, *ttm-1a* and *ttm-1b*, that use different transcription start sites. *ttm-1b* expression was induced by high levels of zinc specifically in intestinal cells, whereas *ttm-1a* was not induced by zinc. TTM-1B was localized to the apical plasma membrane of intestinal cells, and analyses of loss-of-function mutant animals indicated that TTM-1B promotes zinc excretion into the intestinal lumen. Zinc excretion mediated by TTM-1B contributes to zinc detoxification. These observations indicate that *ttm-1* is a component of a negative feedback circuit, since high levels of cytoplasmic zinc increase *ttm-1b* transcript levels and TTM-1B protein functions to reduce the level of cytoplasmic zinc. We showed that TTM-1 isoforms function in tandem with CDF-2, which is also induced by high levels of cytoplasmic zinc and reduces cytoplasmic zinc levels by sequestering zinc in lysosome-related organelles. These findings define a parallel negative feedback circuit that promotes zinc homeostasis and advance the understanding of the physiological roles of the gastrointestinal tract in zinc metabolism in animals.

## Author Summary

Zinc is an essential mineral nutrient involved in many physiological processes, and it plays a critical role in human health. Insufficient dietary zinc causes a wide range of health problems, and excess dietary zinc causes toxicity. Furthermore, genetic mutations affecting zinc metabolism have been implicated in a variety of human diseases. Therefore, animals require homeostatic mechanisms that effectively regulate zinc metabolism in response to dietary fluctuations. The gastrointestinal tract is a major tissue that orchestrates zinc metabolism in animals, and zinc transporters are key molecular regulators involved in this process. To understand these regulatory mechanisms, we used bioinformatic techniques to identify 14 genes that encode predicted Cation Diffusion Facilitator (CDF) family zinc transporters in the *C. elegans* genome. We demonstrated that one of these, *ttm-1*, functions in intestinal cells to promote zinc excretion, and this activity protects animals from zinc toxicity. Genetic analysis revealed that zinc excretion mediated by TTM-1B is coordinated with zinc storage mediated by CDF-2, and these transporters function in a parallel negative feedback circuit to maintain zinc homeostasis in intestinal cells. These findings provide molecular and physiological insight into the regulatory mechanisms of zinc metabolism in animals.

## Introduction

The trace element zinc is essential for all biological systems. Zinc is a well established structural and enzymatic cofactor required for the function of numerous proteins [1], and emerging evidence indicates that zinc functions as a signaling molecule in several biological processes, including development, immune response and synaptic transmission [2]–[4]. Organisms have evolved mechanisms that control zinc homeostasis and metabolism in individual cells and the entire body, and these mechanisms are critical for human health. Diets that contain deficient or excess zinc and result in impaired zinc homeostasis cause a wide range of defects in human health [5], [6]. Furthermore, genetic mutations that cause aberrant zinc metabolism are implicated in a variety of human diseases, such as cancer, diabetes and neurodegenerative diseases [7]–[13]. Thus, understanding mechanisms of zinc homeostasis has important implications for human health.

Zinc metabolism is regulated at the cellular and organismal levels. In eukaryotic cells, the movement of zinc ions across membranes is mediated by transmembrane zinc transporter proteins from two major families, Cation Diffusion Facilitator (CDF/ZnT/SLC30) and Zrt-, Irt-like protein (ZIP/SLC39) [14]. CDF proteins have six conserved transmembrane motifs (TMs) and transport cytoplasmic zinc out of the cell or into the lumen of intracellular organelles, thereby decreasing the level of cytoplasmic zinc. By contrast, ZIP proteins have eight conserved TMs and transport zinc in the opposite directions, thereby increasing the level of cytoplasmic zinc. It appears that almost all zinc ions in the cytoplasm are bound to small molecules or soluble proteins such as metallothioneins [15]. Metallothioneins are small, cysteine-rich proteins that bind up to seven zinc atoms and may function in zinc detoxification and storage. A critical question is how the activity of zinc transporters and metallothioneins are coordinately regulated to maintain zinc homeostasis. Some evidence indicates that, in response to high zinc conditions, the expression of specific CDF proteins and methallothioneins are induced to promote zinc excretion and sequestration in intracellular organelles and proteins, thereby protecting from zinc toxicity [16], [17].

At the organismal level, the gastrointestinal tract is the major site of zinc metabolism that mediates dietary zinc uptake and distribution to the body [18], [19]. In humans, dietary zinc is absorbed into enterocytes across the apical plasma membrane by the action of ZIP4 [13]. Loss-of-function mutations of ZIP4 cause the recessive genetic disease acrodermatitis enteropathica, which is characterized by symptoms of zinc deficiency [8]. ZIP4 expression is highly responsive to dietary zinc levels, which regulates zinc influx into enterocytes [20]. Zinc absorbed in enterocytes is transported by ZnT1 across the basolateral plasma membrane to allow distribution to other tissues [21]. ZnT1 expression is also responsive to dietary zinc levels and induced in high zinc conditions [21], [22]. ZnT1 is essential for viability, since ZnT1-deficient mice display embryonic lethality [23]. Zinc excretion is not as well characterized as zinc absorption. Zinc is excreted in the feces, and a major source of fecal zinc is pancreatic and biliary secretion of zinc containing enzymes [18]. However, the primary role of these enzymes is likely to be digestion rather than zinc homeostasis. The role of enterocytes in zinc excretion via zinc transporters is only beginning to emerge. The ZnT5 variant B is localized to the apical membrane of enterocytes and is reported to function in both zinc efflux and influx [24], [25]. Additional zinc transporters are localized to intracellular compartments in enterocytes. For example, ZnT5A, ZnT6, ZnT7 and ZIP7 are localized to the Golgi complex, and ZnT2 and ZnT4 are localized to endosomal or lysosomal vesicles [19]. These proteins might be involved in zinc excretion through the secretory pathway, although *in vivo* evidence for this function has not been well established.

The nematode *C. elegans* is a useful model organism to study zinc metabolism. Facilitated by powerful genetic techniques, studies of *C. elegans* have contributed to the discovery of novel functions of zinc ions in signal transduction during development and to understanding mechanisms that regulate zinc metabolism and homeostasis [26]–[31]. The *C. elegans* genome encodes many evolutionarily conserved proteins involved in zinc metabolism, including members of the CDF, ZIP and methallothionein families [14], [32], indicating that it will be relevant to understanding mechanisms of zinc metabolism in higher animals. Similar to higher animals, intestinal cells of *C. elegans* are the point of zinc entry and the critical tissue for zinc homeostasis. CDF-1 and SUR-7 are expressed in intestinal cells and localized to the plasma membrane and the ER/Golgi complex, respectively [26], [31]. Lysosome-related organelles in intestinal cells, known as gut granules, function as a physiological zinc storage site that is important for zinc detoxification and mobilization in response to fluctuating dietary zinc levels, and CDF-2 plays an essential role in this process [30]. Furthermore, the metallothionein genes *mtl-1* and *mtl-2* are highly expressed in intestinal cells [32]. Therefore, the intestine of *C. elegans* appears to function as a major organ that controls zinc metabolism and may be a relevant model for the gastrointestinal tract in mammals.

Here we identified 14 predicted CDF family members encoded by the *C. elegans* genome using iterative sequence based homology search methods. We focused on *ttm-1 (toxin-regulated target of p38MAPK)*, because the predicted TTM-1 proteins are highly related to vertebrate CDF proteins and the role of this gene in zinc metabolism had not been characterized. *ttm-1* was initially identified as a downstream target of p38 MAP kinase that is induced in response to pore-forming bacterial toxins [33]. In addition, *ttm-1* was reported to be induced by cadmium exposure [34]. To elucidate the function of *ttm-1* in zinc metabolism, we characterized the *ttm-1* gene structure, expression and function. *ttm-1* encodes two protein isoforms: TTM-1A was not zinc regulated, whereas TTM-1B was induced by high levels of dietary zinc. Each isoform displayed unique cellular and subcellular expression patterns, and TTM-1B displayed a striking subcellular localization at the apical plasma membrane of intestinal cells. Using a zinc-specific fluorescent dye to visualize zinc and quantitative zinc level analyses, we showed that *ttm-1* functions in zinc excretion from intestinal cells into the lumen of the gut. Zinc excretion mediated by TTM-1B was coordinated with zinc storage in gut granules mediated by CDF-2, and these two genes function in a parallel negative feedback circuit to promote zinc detoxification and homeostasis. These studies rigorously document zinc excretion from intestinal cells mediated by a CDF protein and the coordinated action of multiple CDF proteins in zinc detoxification, and may be relevant to understanding zinc metabolism in higher animals.

## Results

### Identification of predicted *C. elegans* CDF family members

**Figure 1:**
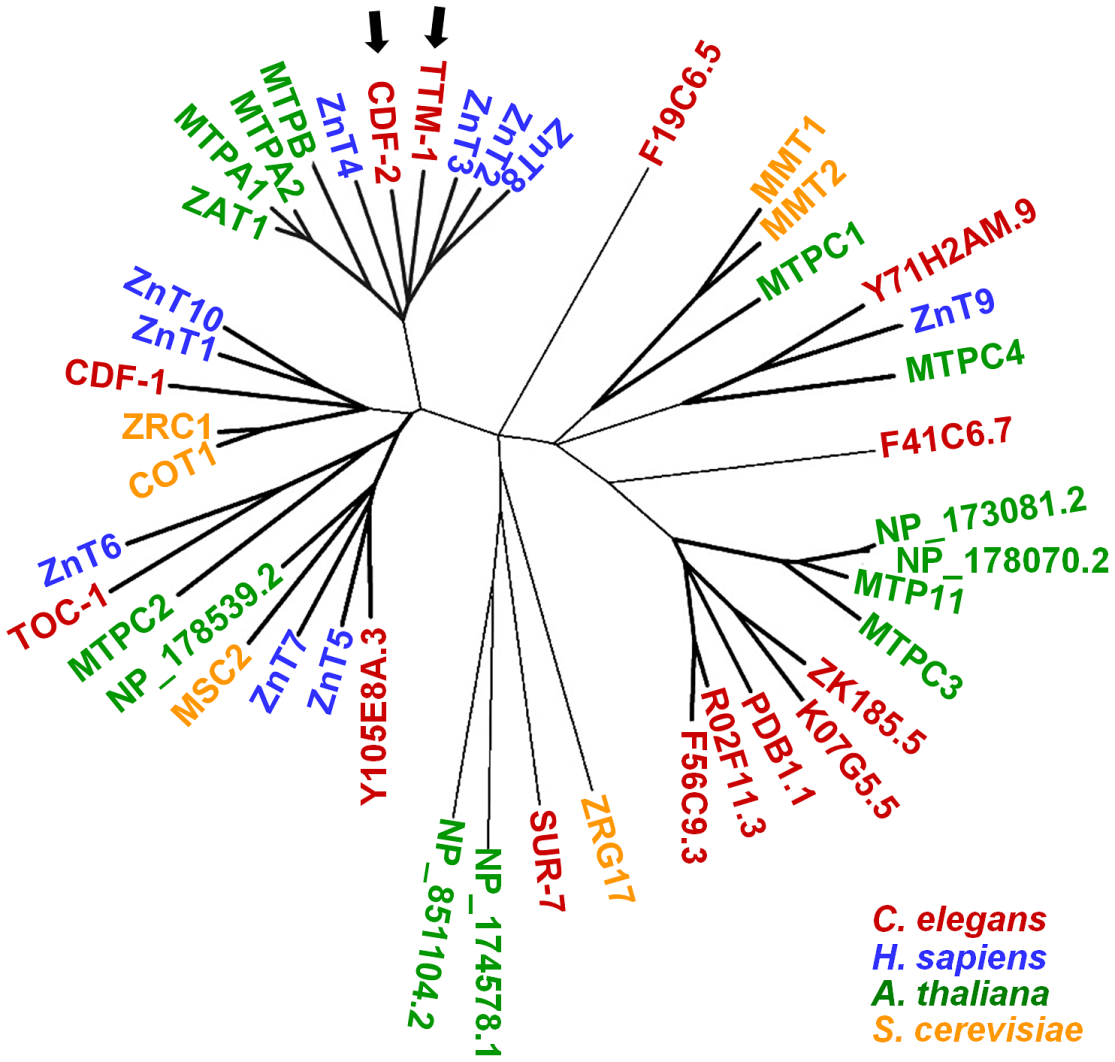
Phylogenetic tree of CDF family members. A dendrogram showing 14 predicted *C. elegans* CDF family members (red) identified by PSI-BLAST and all predicted CDF proteins from *Homo sapiens* (blue), *Arabidopsis thaliana* (green), and the yeast *Saccaromyces cerevisiae* (yellow). TTM-1 and CDF-2 are indicated by arrows. Notably, all ten human ZnT proteins cluster with highly related *C. elegans* proteins. For genes that encode multiple protein isoforms, only one isoform is listed.

To identify *C. elegans* CDF proteins, we employed iterative sequence-based homology search methods using the Position-Specific Iterated BLAST (PSI-BLAST) [35]. Beginning with the protein sequence of CDF-1, a total of 14 *C. elegans* proteins were identified, including SUR-7 and CDF-2 that were previously characterized and eleven additional CDF proteins (Figure 1). To analyze evolutionary conservation of CDF proteins, we compared the 14 *C. elegans* proteins with CDF proteins of yeast, plants and humans using the reciprocal PSI-BLAST. This analysis generated a phylogenetic tree of CDF proteins and suggested that CDF proteins have diverged into several subfamilies (Figure 1). Human ZnT proteins were present in four clusters, and all of these clusters contained *C. elegans* proteins, suggesting that *C. elegans* contains CDF proteins that may be relevant to the function of most or all human CDF proteins. There were also clusters that contained *C. elegans* proteins but no human proteins and clusters that contained only plant and yeast proteins, suggesting that some CDF proteins in yeast, plants and worms have functions not represented in humans.

### Gene and protein structure of *ttm-1*

**Figure 2:**
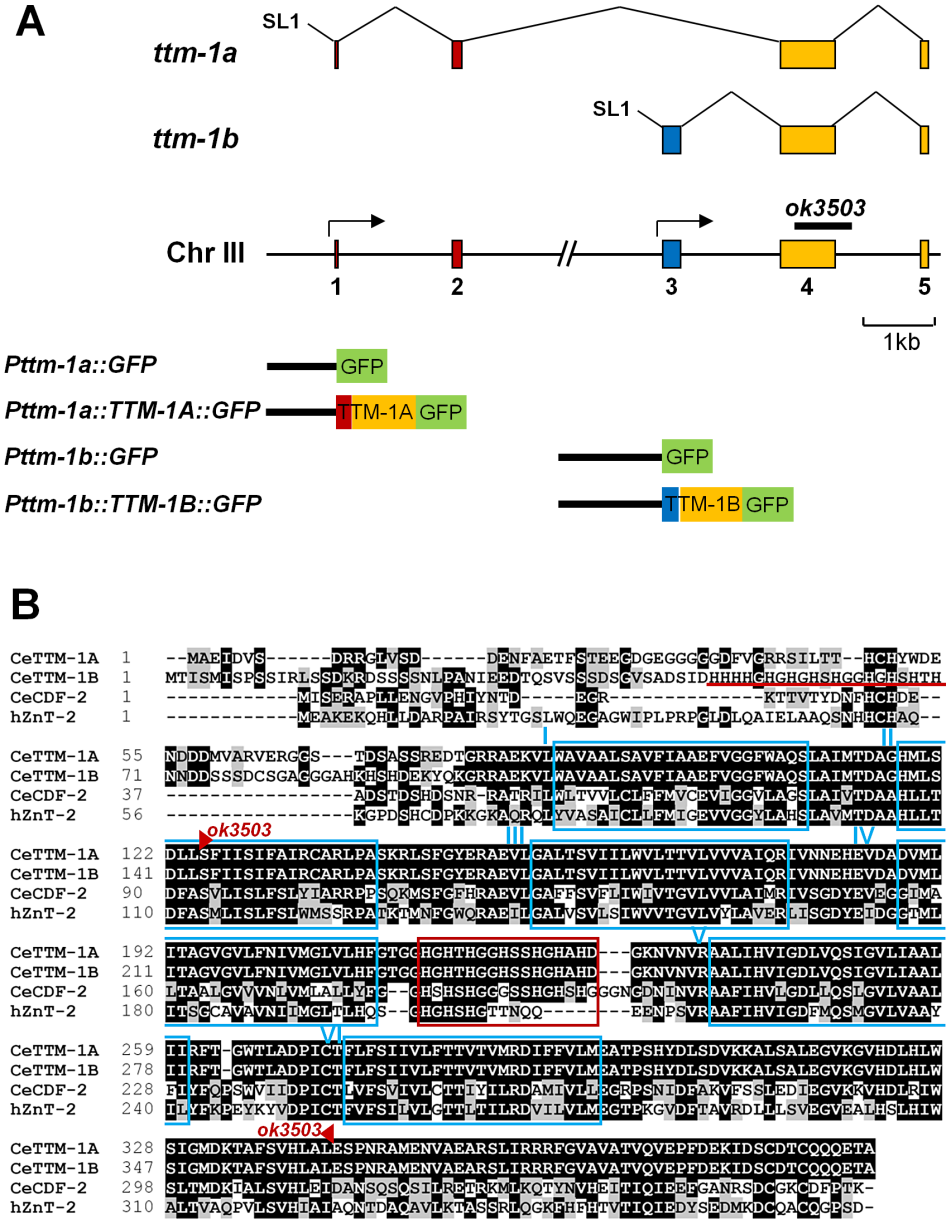
Structure of the *ttm-1* gene and protein. (A) The straight line indicates genomic DNA on chromosome III: numbered boxes indicate exons, and arrows indicate transcription start sites. The splicing patterns of *ttm-1a* and *ttm-1b* are shown above: Exons 1 and 2 (red) are unique to *ttm-1a*, exon 3 (blue) is unique to *ttm-1b*, and exons 4 and 5 (yellow) are common to both transcripts. SL1 indicates trans-spliced leader sequence. The black bar denotes the genomic region deleted in the *ok3503* allele. Constructs used for expression pattern analysis are depicted below. Black lines denote promoter regions, and colored boxes denote protein coding sequences. (B) An alignment of the predicted TTM-1A and TTM-1B proteins with *C. elegans* CDF-2 and human ZnT2. Identical and similar amino acids are highlighted in black and gray, respectively. Blue boxes indicate the predicted six transmembrane motifs (labeled I–VI). The red box indicates the conserved histidine-rich motif, (HX)_n_, and the red bar indicates the additional histidine-rich motif in TTM-1B. Red triangles flank the amino acids encoded by the region deleted in the *ok3503* allele.

We focused on *ttm-1* because it is highly related to human ZnT2, 3, 4 and 8 (Figure 1). *ttm-1* expression was previously analyzed, since it is induced by pore-forming bacterial toxins and cadmium exposure [33], [34], but the function of *ttm-1* has not been reported. The computational algorithm Gene Finder and the analysis of cDNAs indicated that *ttm-1* consists of five exons and encodes two transcripts; *ttm-1a* is generated by splicing exons 1, 2, 4, and 5, whereas *ttm-1b* is generated by splicing exons 3, 4, and 5 (Figure 2A). To determine if the two transcripts result from alternative splicing of a single primary transcript or alternative transcription initiation sites, we used 5′ rapid amplification of cDNA ends (5′ RACE). If the two transcripts were generated by alternative processing of the same precursor, then *ttm-1a* and *ttm-1b* are predicted to contain SL1 and SL2 trans-spliced leader sequences at the 5′ends, respectively. By contrast, if *ttm-1a* and *ttm-1b* are independently transcribed, then both are predicted to contain the SL1 trans-spliced leader sequence [36]. The SL1 trans-spliced leader sequence was detected at the 5′ end of both *ttm-1a* and *ttm-1b* transcripts (Figure S1). These results indicate that the *ttm-1* locus has two transcription initiation sites that generate two primary transcripts that are spliced to form *ttm-1a* and *ttm-1b* (Figure 2A).

The predicted TTM-1A and TTM-1B proteins have the signature conserved motifs of the CDF family: six transmembrane motifs (TMs) and a conserved histidine-rich motif, (HX)_n_, in the loop between TM IV and V (Figure 2B). The (HX)_n_ motif is implicated in zinc binding and regulation of transport activity [37]. TTM-1A and TTM-1B showed the highest sequence similarity with CDF-2 from *C. elegans* and ZnT2 from humans (Figure 2B). As a result of different transcription start sites, TTM-1A and TTM-1B have distinct amino acid sequences at N-terminus. TTM-1B has an additional histidine-rich motif in the N-terminus, which is similar to the (HX)_n_ motif in the loop between TM IV and V (Figure 2B), suggesting that TTM-1B may have an N-terminal zinc-binding domain that influences transporter function or regulation by zinc.

### Expression pattern of *ttm-1*

**Figure 3:**
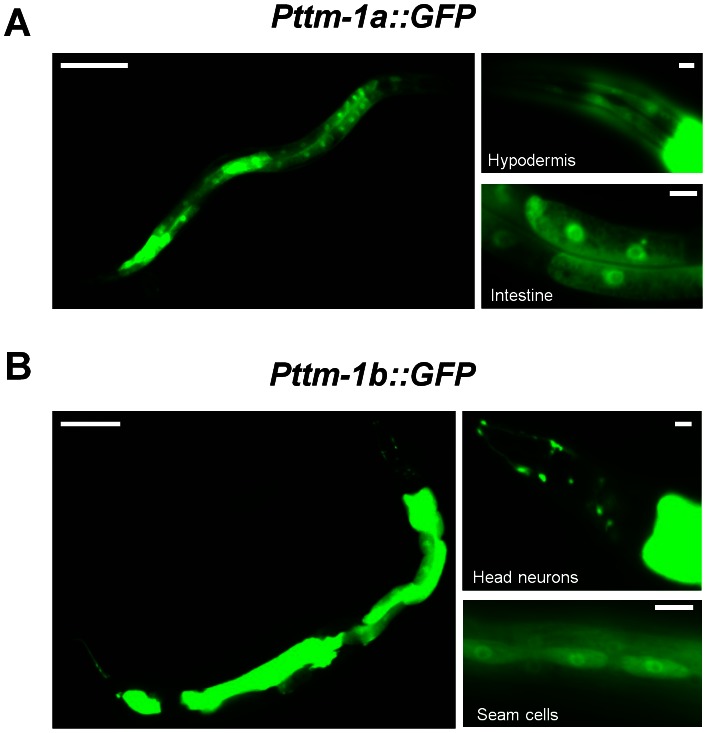
Expression pattern of *ttm-1*. Fluorescence microscope images of live transgenic hermaphrodites expressing GFP under the control of (A) the predicted *ttm-1a* promoter [*Pttm-1a::GFP*] and (B) the predicted *ttm-1b* promoter [*Pttm-1b::GFP*]. The left panel shows an entire worm, and both promoters are expressed in intestinal cells. Right panels are magnified views of hypodermal and intestinal expression driven by the *ttm-1a* promoter and head neuron and seam cell expression driven by the *ttm-1b* promoter. To visualize the relatively weak GFP fluorescence in head neurons, we used a longer exposure time compared to Figure 4B. Scale bars represent 100 µm (left) and 10 µm (right).

To determine cell types that express *ttm-1*, we generated plasmids that express green fluorescent protein (GFP) under the control of the predicted *ttm-1a* and *ttm-1b* promoters, introduced these constructs into transgenic animals, and monitored fluorescence. For *ttm-1a*, the promoter region consisted of ∼1.2 kb of genomic DNA that extends from the *ttm-1a* start codon upstream to the 3′ end of the adjacent gene (Figure 2A). *Pttm-1a::GFP* transgenic animals displayed GFP expression in the hypodermis and the intestine (Figure 3A). For *ttm-1b*, the promoter region consisted of ∼6 kb of genomic DNA that extends from the *ttm-1b* start codon in exon 3 upstream into intron 2 (Figure 2A). *Pttm-1b::GFP* transgenic animals displayed GFP expression in multiple tissues including the intestine, head neurons, seam cells, hypodermis and the vulva (Figure 3B). In both cases, similar patterns were displayed by multiple, independently derived transgenic strains, indicating these results are reproducible. These results indicate that both *ttm-1* transcripts are expressed in intestinal cells, and each has a distinct expression pattern in non-intestinal cells.

### *ttm-1b* expression is regulated by dietary zinc

**Figure 4:**
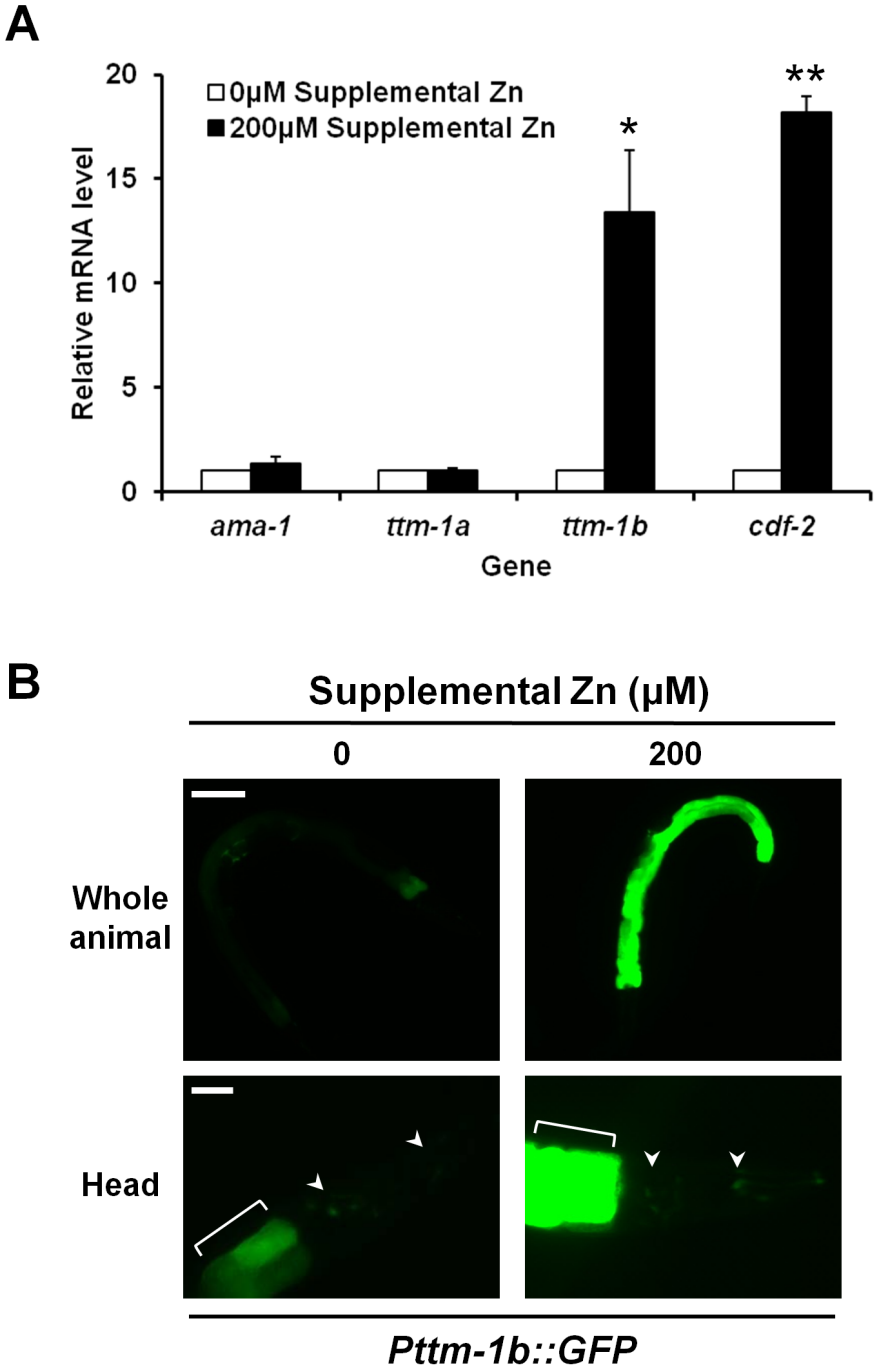
Regulation of *ttm-1* expression by zinc. (A) Wild-type animals were cultured with 0 µM or 200 µM supplemental zinc. RNA was extracted from a synchronized population at L4/adult stages, and mRNA levels of indicated genes were determined by quantitative real-time PCR. The Y-axis represents the fold changes of mRNA levels between 0 µM and 200 µM supplemental zinc, and the bars indicate the average ± SEM of three independent experiments. The mRNA levels at 0 µM supplemental zinc were set equal to 1.0 for each gene. *ttm-1b* was significantly induced by 200 µM compared to 0 µM supplemental zinc (*p<0.05), similar to the positive control gene *cdf-2* (**p<0.01) [28], [30]. *ttm-1a* was not responsive to zinc, similar to the negative control gene *ama-1* [28], [30]. (B) Fluorescence microscope images of *Pttm-1b::GFP* transgenic animals cultured with 0 µM or 200 µM supplemental zinc. Whole animals are shown in the top panels, and the head and anterior most intestinal cells are shown in the bottom panels (anterior to the right). While GFP expression was induced in intestinal cells by 200 µM supplemental zinc (top panel and brackets in lower panel), GFP expression was not induced in the head neurons (arrow heads). Images in each panel were captured with the identical settings and exposure times. Scale bars represent 100 µm (top) and 20 µm (bottom).

To determine whether the expression of *ttm-1* is regulated by dietary zinc levels, we cultured wild-type animals on noble agar minimal media (NAMM) containing 0 µM or 200 µM supplemental zinc [27] and analyzed mRNA levels using quantitative real time PCR (RT-PCR). The level of *ttm-1a* mRNA was not affected by dietary zinc, similar to the control gene *ama-1* which encodes the large subunit of RNA polymerase II and is not regulated by dietary zinc [28]. By contrast, the level of *ttm-1b* mRNA was significantly elevated by supplemental dietary zinc, similar to the zinc-inducible gene *cdf-2* [28], [30] (Figure 4A). These results indicate that dietary zinc increases the synthesis and/or decreases the degradation of *ttm-1b* mRNA.

To determine how *ttm-1b* expression is regulated in different tissues in response to high levels of dietary zinc, we analyzed *Pttm-1b::GFP* transgenic animals. *Pttm-1b::GFP* transgenic animals cultured with 200 µM supplemental zinc displayed increased GFP expression in intestinal cells (Figure 4B, top). However, GFP expression was not increased in the other tissues, such as head neurons (Figure 4B, bottom). Thus, the 6 kb *ttm-1b* promoter fragment contains regulatory elements that are sufficient to mediate zinc-responsive transcriptional induction in intestinal cells, suggesting regulation occurs at the level of transcriptional initiation. Furthermore, *ttm-1b* expression appears to be differentially regulated by dietary zinc in different cell types.

### Intracellular localization of TTM-1 isoforms

**Figure 5:**
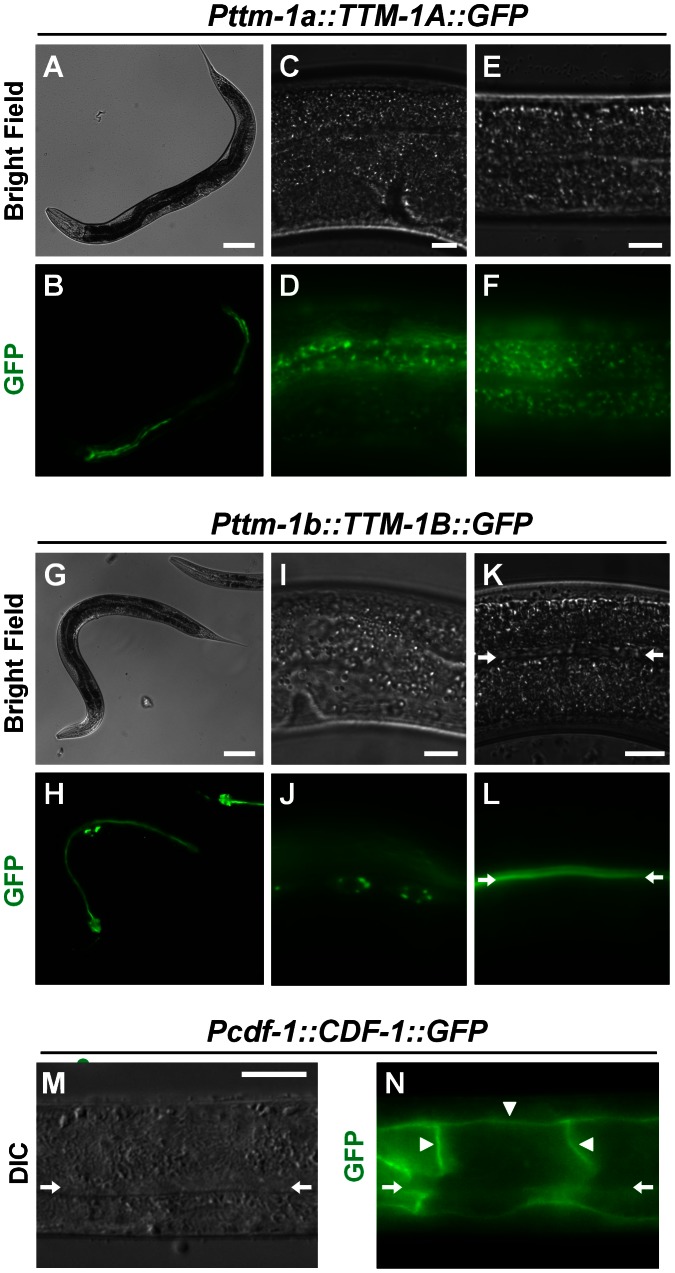
Intracellular localization of TTM-1 isoforms and CDF-1. Bright field and fluorescence microscope images of live transgenic animals expressing TTM-1A::GFP [*Pttm-1a::TTM-1A::GFP*] (A–F) or TTM-1B::GFP [*Pttm-1b::TTM-1B::GFP*] (G–L). Differential interference contrast (DIC) and fluorescence microscope images of a fixed transgenic animal expressing CDF-1::GFP [*Pcdf-1::CDF-1::GFP*] that was immunostained with an anti-GFP antibody (M–N). Bright field and DIC show morphology, and green displays GFP. Images show an entire animal (A–B, G–H), intestinal cells (C–D, K–L, M–N), hypodermal cells (E–F), and seam cells (I–J). The arrows indicate the lumen of the intestine (K–N). TTM-1A::GFP displayed diffuse, punctuate staining in intestinal and hypodermal cells. TTM-1B::GFP displayed punctuate staining in seam cells and was localized to the apical plasma membrane of intestinal cells. The diffuse fluorescence signal in panel J derives from GFP that is out of the focal plane. CDF-1::GFP was localized to the basolaternal plasma membrane of intestinal cells (triangles). Scale bars represent 100 µm (A, G) and 10 µm (C, E, I, K, M).

To analyze the intracellular localizations of TTM-1A and TTM-1B proteins, we generated plasmids that encode full length TTM-1A or TTM-1B protein fused to GFP under the control of the corresponding promoter (Figure 2A) and introduced these plasmids into transgenic animals. TTM-1A::GFP was expressed in the intestine and hypodermis (Figure 5A–5F); the same tissue distribution pattern was displayed by transgenic animals expressing GFP alone under the control of the *ttm-1a* promoter, indicating that the coding sequences do not substantially influence the tissue expression pattern. In intestinal and hypodermal cells, TTM-1A::GFP displayed a punctuate pattern (Figure 5C–5F). TTM-1A::GFP did not colocalize with the autofluorescence of gut granules in intestinal cells or with MitoTracker in hypodermal cells (data not shown), suggesting that TTM-1A does not localize to lysosome-related organelles or mitochondria.

TTM-1B::GFP was expressed in multiple tissues including intestine, head neurons and seam cells (Figure 5G–5L), consistent with the tissue distribution pattern displayed by *Pttm-1b::GFP* transgenic animals. TTM-1B::GFP was localized to intracellular compartments in several tissues such as seam cells (Figure 5I, 5J) and head neurons (data not shown). However, in intestinal cells, TTM-1B::GFP was localized to the plasma membrane and restricted to the apical side of the cell which forms the surface of the intestinal lumen (Figure 5K, 5L). Thus, TTM-1B displayed distinct intracellular localization in different cell types.

CDF-1 is localized to the plasma membrane of intestinal cells [26]. To compare the localization of CDF-1 with TTM-1B, we immunostained transgenic animals expressing CDF-1::GFP with an anti-GFP antibody to amplify GFP signal in intestinal cells and eliminate autofluorescence from gut granules. CDF-1::GFP was detected on the plasma membrane where it was restricted to the basolaternal surface of intestinal cells that face the body cavity and absent from the apical surface (Figure 5M, 5N). These results suggest that TTM-1B and CDF-1 are localized in a non-overlapping pattern on the apical and basolateral plasma membranes of intestinal cells, respectively.

### Role of *ttm-1* in zinc excretion

To characterize the function of *ttm-1*, we obtained the mutant allele *ttm-1(ok3503)* that was generated by the *C. elegans* Gene Knockout Project at Oklahoma Medical Research Foundation (OMRF). By analyzing the genomic DNA sequence of this allele, we determined that the *ok3503* mutation is a deletion of 877 bp beginning in exon 4 and extending to intron 4 (Figure 2A and Figure S2). This deletion removes the coding sequence for part of TM II, all of TM III, IV, V and VI, and the (HX)_n_ motif (Figure 2B). Because these motifs are highly conserved in the CDF family, the *ok3503* mutation is likely to severely disrupt the activity of TTM-1 proteins. The deleted region affects both TTM-1A and TTM-1B (Figure 2A), suggesting that *ttm-1(ok3503)* is a strong loss-of-function mutation that reduces the activity of both isoforms. To determine the effect of the *ok3503* mutation on transcription of *ttm-1*, we analyzed *ttm-1a* and *ttm-1b* mRNA levels by RT-PCR. Both *ttm-1* transcripts were detected in the *ttm-1(ok3503)* strain, and *ttm-1b* expression was elevated in response to high levels of dietary zinc, similar to wild-type animals (data not shown). Thus, the *ttm-1(ok3503)* allele does not appear to affect transcription initiation.

**Figure 6:**
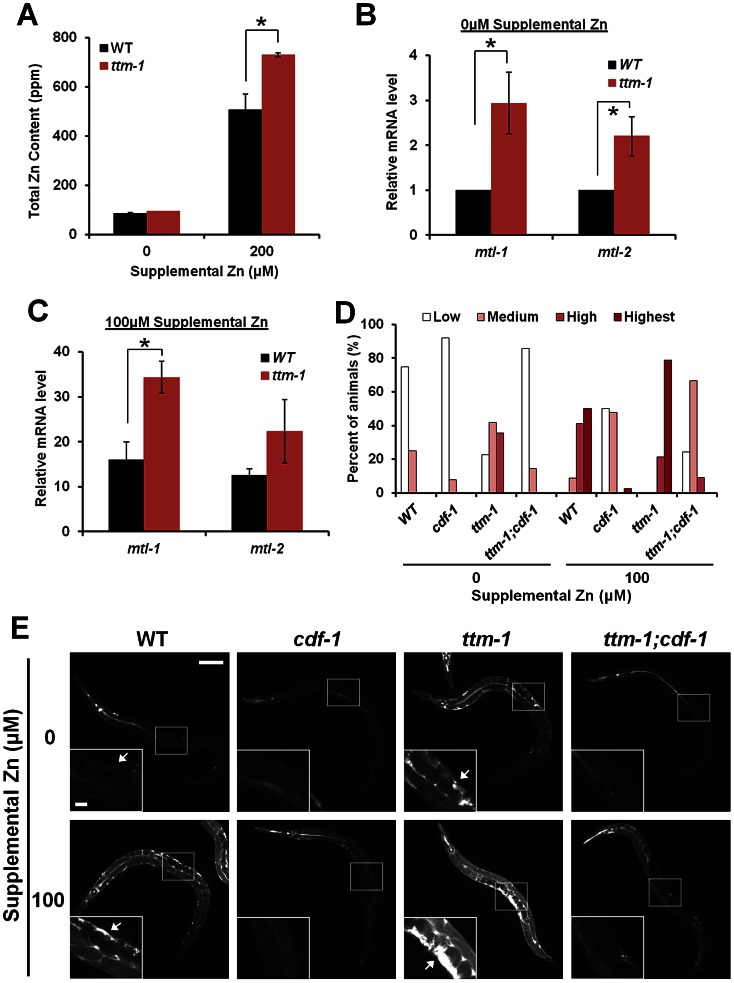
*ttm-1* functions in zinc excretion. (A) Total zinc content of wild-type and *ttm-1(ok3503)* animals. Populations consisting of mixed developmental stages were cultured with the indicated concentrations of supplemental zinc and analyzed for total zinc content by ICP-MS. Total zinc content was calculated in parts-per-million (ppm) by dividing the weight of zinc by the weight of dessicated worms (µg/g). Bars indicate average ± SEM of two independent experiments (*p<0.05). (B–C) RT-PCR analysis of mRNA levels of *mtl-1* or *mtl-2* in wild-type and *ttm-1(ok3503)* animals cultured with 0 µM or 100 µM supplemental zinc. The Y-axis represents the fold changes of mRNA levels, and the bars indicate the average ± SEM of two independent experiments. Wild-type animals cultured in 0 µM supplemental zinc were set equal to 1.0, and the other samples were relative to that sample (*p<0.05). (D–E) Fluorescence microscope images of live wild-type, *cdf-1(n2527), ttm-1(ok3503)*, and *ttm-1(ok3503);cdf-1(n2527)* animals stained with Zinpyr-1. Animals were cultured with 0 µM or 100 µM supplemental zinc – a concentration that causes zinc accumulation without causing excessive toxicity. Images show whole animals, and insets show magnified images of the boxed regions. Arrows indicate strong Zinpyr-1 staining in the pseudoceolomic space around the uterus. The scale bars represent 100 µm and 20 µm (insets). To quantify Zinpyr-1 staining, we categorized animals into four groups based on Zinpyr-1 fluorescence intensity: highest (dark red), high (red), medium (pink) and low (white). The Y-axis represents the percent of each group in the population (n≥20). The quantification was repeated twice with similar results, and panel D shows representative data from one experiment.

To determine the role of *ttm-1* in zinc metabolism, we measured total zinc content of worms using inductively coupled plasma-mass spectrometry (ICP-MS). With 0 µM supplemental zinc, wild-type and *ttm-1(ok3503)* mutant animals displayed similar total zinc content. In the presence of 200 µM supplemental zinc, *ttm-1* mutant animals displayed approximately 40% higher total zinc content than wild-type animals (Figure 6A). Thus, *ttm-1* may promote zinc excretion in high levels of dietary zinc. Expression levels of metallothionein genes can be regulated by a variety of stressors, such as heavy metals and oxidative damage, and metallothionein gene expression is upregulated by high levels of environmental zinc [38]. To indirectly measure zinc levels, we analyzed *mtl-1* and *mtl-2* mRNA levels using RT-PCR. Both wild type and *ttm-1* mutant animals displayed a substantial increase in the levels of *mtl-1* and *mtl-2* transcripts when exposed to 100 µM supplemental zinc (Figure 6B, 6C), demonstrating that *C. elegans* metallothionein genes can be induced by dietary zinc. Furthermore, in the presence of 0 µM and 100 µM supplemental zinc, *ttm-1* mutant animals displayed approximately 4-fold and 2-fold higher levels of *mtl-1* mRNA, respectively, compared to wild-type animals (Figure 6B, 6C). Similar results were observed for *mtl-2* mRNA expression (Figure 6B, 6C). Together, these results indicate that zinc hyperaccumulates in *ttm-1* mutant animals, suggesting that TTM-1 isoforms function in the excretion of zinc out of the body.

Zinc-responsive fluorescent dyes have been used to visualize the distribution of zinc in various model systems, and we previously reported that FluoZin-3 specifically detects labile zinc stored in gut granules in *C. elegans* [30]. To visualize zinc in other locations in *C. elegans*, we analyzed additional zinc-responsive fluorescent dyes and discovered that wild-type animals incubated with the dye Zinpyr-1 displayed fluorescence primarily in the pseudocoelom (Figure 6E). Animals cultured with 100 µM supplemental zinc displayed elevated Zinpyr-1 fluorescence in the pseudocoelom compared to 0 µM supplemental zinc (Figure 6D, 6E), suggesting that Zinpyr-1 fluorescence is a measure of labile zinc and the level of pseudocoelomic zinc is responsive to dietary zinc levels. The pseudocoelom is a fluid-filled space that interfaces with the basolateral surface of intestinal cells and epithelial cells and surrounds internal organs, such as the gonad, muscles and neurons. The fluid in the pseudocoelom is likely to transport nutrients such as zinc from the intestinal cells to other tissues, and Zinpyr-1 appears to visualize this pool of labile zinc.

To analyze the regulation of pseudocoelomic zinc, we examined *cdf-1* mutant animals. CDF-1 was localized to the basolateral surface of the plasma membrane of intestinal cells, the interface between the intestine and pseudocoelom (Figure 5M, 5N), and thus we predicted that reducing CDF-1 activity might decrease zinc levels in the pseudocoelom. *cdf-1(n2527)* is a strong loss-of-function mutation that causes a nonsense change [26]. *cdf-1* mutant animals displayed decreased Zinpyr-1 fluorescence in the pseudocoelom compared to wild-type animals, and the difference was striking in 100 µM supplemental zinc (Figure 6D, 6E). These results suggest that Zinpyr-1 specifically detects zinc in the pseudocoelom and CDF-1 plays a key role in transporting zinc from intestinal cells to the pseudocoelom.

*ttm-1* mutant animals displayed elevated Zinpyr-1 fluorescence in the pseudocoelom compared to wild-type animals (Figure 6D, 6E). Thus, the pseudocoelom is one compartment that hyperaccumulates zinc in *ttm-1* mutant animals. To investigate the relationship between TTM-1 isoforms and CDF-1, we analyzed *ttm-1;cdf-1* double mutant animals. Double mutant animals displayed a low level of Zinpyr-1 fluorescence in the pseudocoelom, similar to *cdf-1* single mutant animals (Figure 6D, 6E). These results suggest that the accumulation of zinc in the pseudocoelom displayed by *ttm-1* mutant animals requires the activity of CDF-1. We propose that TTM-1 isoforms function upstream of CDF-1 to reduce zinc levels in the pseudocoelom by localizing to the apical surface of the plasma membrane of intestinal cells and functioning to excrete zinc. In *ttm-1* mutant animals, excess zinc in the cytoplasm of intestinal cells is transported into the pseudocoelom by CDF-1.

### *ttm-1* functions in zinc detoxification

**Figure 7:**
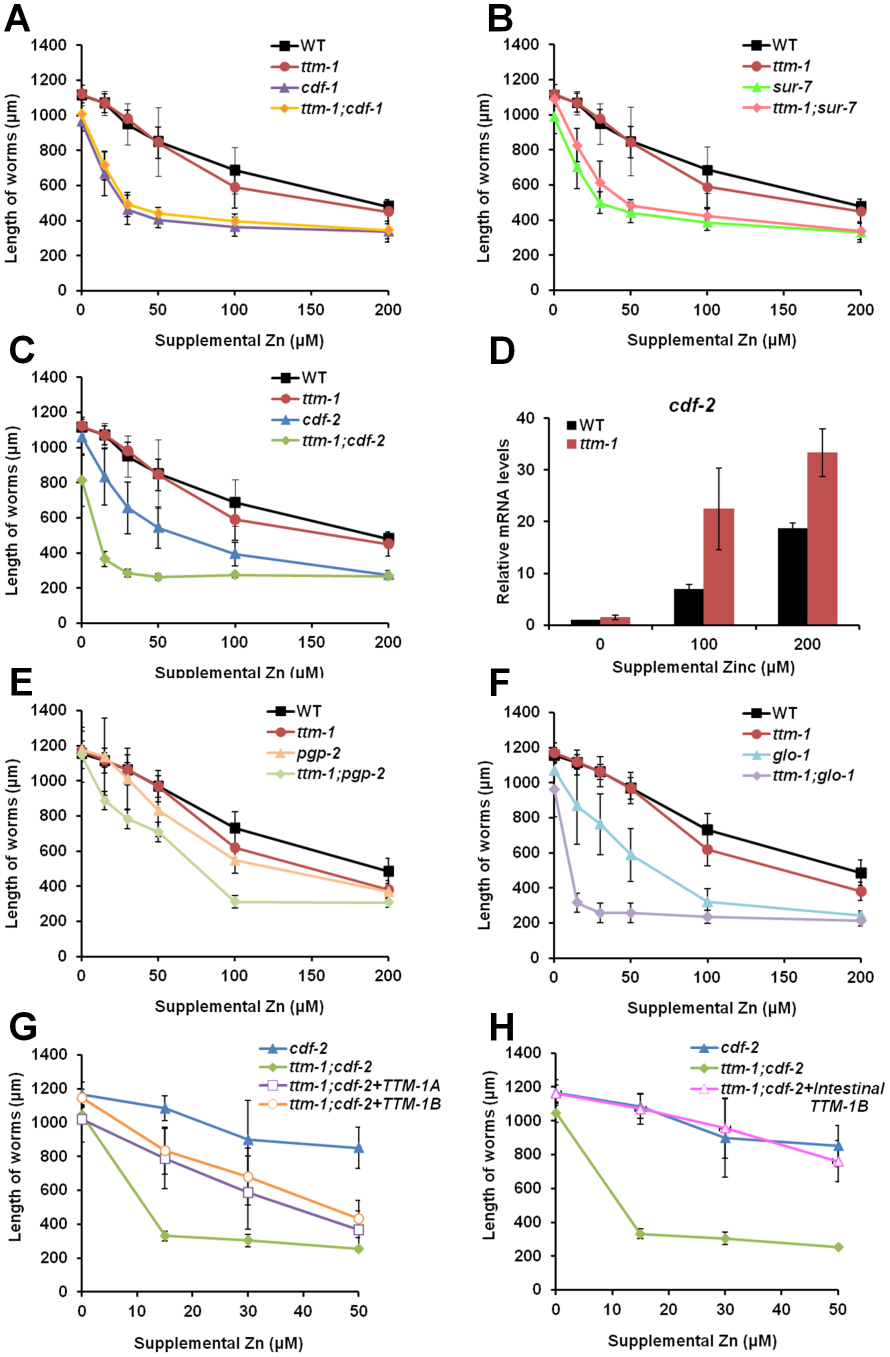
Role of *ttm-1* in zinc detoxification. (A,B,C,E,F) Zinc sensitivity was determined by analyzing the length of animals cultured from the first larval (L1) stage with the indicated concentrations of supplemental zinc. The length of worms was analyzed using microscopy and imageJ software (n = 20). The alleles were *ttm-1(ok3503)*, *cdf-1(n2527)*, *sur-7(ku119)*, *cdf-2(tm788)*, *glo-1(zu391)*, and *pgp-2(kx48)*. (D) Analysis of *cdf-2* mRNA levels in wild-type and *ttm-1(ok3503)* animals cultured with the indicated concentrations of supplemental zinc. The Y-axis represents the fold changes of mRNA levels, and the bars indicate the average ± SEM of two independent experiments. The mRNA level in wild-type animals at 0 µM supplemental zinc was set equal to 1.0, and the other samples were relative to that sample. Compared to wild-type animals, *cdf-2* mRNA levels were elevated in *ttm-1* mutant animals cultured with supplemental zinc in both independent trials, indicating this is a reproducible result. However, the combined data did not reach statistical significance at the level of p<0.05 because the values of the fold changes varied between the experiments. (G–H) Zinc sensitivity of *cdf-2(tm788)*, *ttm-1(ok3503);cdf-2(tm788)*, and transgenic *ttm-1(ok3503);cdf-2(tm788)* animals expressing TTM-1A::GFP driven by the *ttm-1a* promoter [*Pttm-1a::TTM-1A::GFP*] (G), TTM-1B::GFP driven by the *ttm-1b* promoter [*Pttm-1b::TTM-1B::GFP*] (G), or TTM-1B::GFP driven by the *cdf-2* promoter [*Pcdf-2::TTM-1B::GFP*] (H) (n = 20).

To determine if zinc excretion by TTM-1 isoforms contributes to zinc detoxification, we examined zinc sensitivity by measuring the growth rate of animals in the presence of different concentrations of supplemental zinc. In all zinc conditions, *ttm-1* mutant animals displayed similar or marginally reduced growth rates compared to wild-type animals (Figure 7A). To address the possibility that other CDF proteins may function redundantly with TTM-1 isoforms, we examined double mutant animals with *cdf-1(n2527)*, *cdf-2(tm788)* and *sur-7(ku119)*. *cdf-2(tm788)* is a strong loss-of-function mutation caused by a deletion/insertion that disrupts transcription [28], and *sur-7(ku119)* is a partial loss-of-function mutation caused by a base pair change in a splice site [31]. *cdf-1* and *sur-7* mutant animals displayed reduced growth rates in the presence of supplemental zinc compared to wild-type animals (Figure 7A and 7B), consistent with the previous reports that *cdf-1* and *sur-7* mutant animals are hypersensitive to zinc [26], [31]. The zinc sensitivities of *ttm-1;cdf-1* and *ttm-1;sur-7* double mutant animals were similar to *cdf-1* and *sur-7* single mutant animals, respectively (Figure 7A and 7B). By contrast, *ttm-1;cdf-2* double mutant animals displayed strikingly enhanced zinc sensitivity compared to *cdf-2* single mutant animals (Figure 7C), indicating that TTM-1 isoforms have an important function in zinc detoxification in animals that lack CDF-2. To investigate whether CDF-2 activity is altered to compensate for the loss of TTM-1 isoforms, we compared *cdf-2* mRNA levels between wild type and *ttm-1* mutant animals. *cdf-2* mRNA levels were higher in *ttm-1* mutant animals (Figure 7D), suggesting that increased CDF-2 expression is a mechanism that compensates for the loss of *ttm-1* function in zinc detoxification.

CDF-2 mediates zinc storage in gut granules. Glo mutant animals are defective in the biogenesis of gut granules [39], [40] and, therefore, are defective in zinc detoxification [30]. *pgp-2* mutant animals have a moderately reduced number of gut granules compared to wild-type animals [40], and *glo-1* mutant animals have a severely reduced number of gut granules [39]. *ttm-1;pgp-2* double mutant animals displayed enhanced zinc sensitivity compared to *pgp-2* single mutant animals (Figure 7E). *ttm-1;glo-1* double mutant animals displayed substantially enhanced zinc sensitivity compared to *glo-1* single mutant animals (Figure 7F). These results are consistent with the analysis of CDF-2 and suggest that TTM-1 isoforms function in zinc detoxification in cooperation with zinc storage in gut granules.

To determine the role of TTM-1 isoforms in zinc detoxification, we generated transgenic animals that express TTM-1A::GFP or TTM-1B::GFP. Transgenic *ttm-1;cdf-2* mutant animals expressing either TTM-1A::GFP or TTM-1B::GFP displayed increased growth rates compared to *ttm-1;cdf-2* mutant animals (Figure 7G), suggesting that both TTM-1A::GFP and TTM-1B::GFP isoforms are functional zinc transporters and contribute to zinc detoxification. To further analyze the site of action, we generated transgenic animals expressing TTM-1B::GFP under the control of *cdf-2* promoter (Figure S3). In these animals, TTM-1B::GFP is expressed specifically in intestinal cells and is zinc inducible. Intestine-specific expression of TTM-1B::GFP in *ttm-1;cdf-2* double mutant animals rescued zinc sensitivity to the level of *cdf-2* single mutant animals (Figure 7H). Thus, TTM-1B expression in intestinal cells was sufficient to rescue the zinc hypersensitivity phenotype of *ttm-1;cdf-2* mutant animals. These results suggest that TTM-1B localized to the apical surface of the plasma membrane of intestinal cells plays a critical role in zinc excretion and detoxification.

### *ttm-1* and *cdf-2* function coordinately to control cytoplasmic zinc

**Figure 8:**
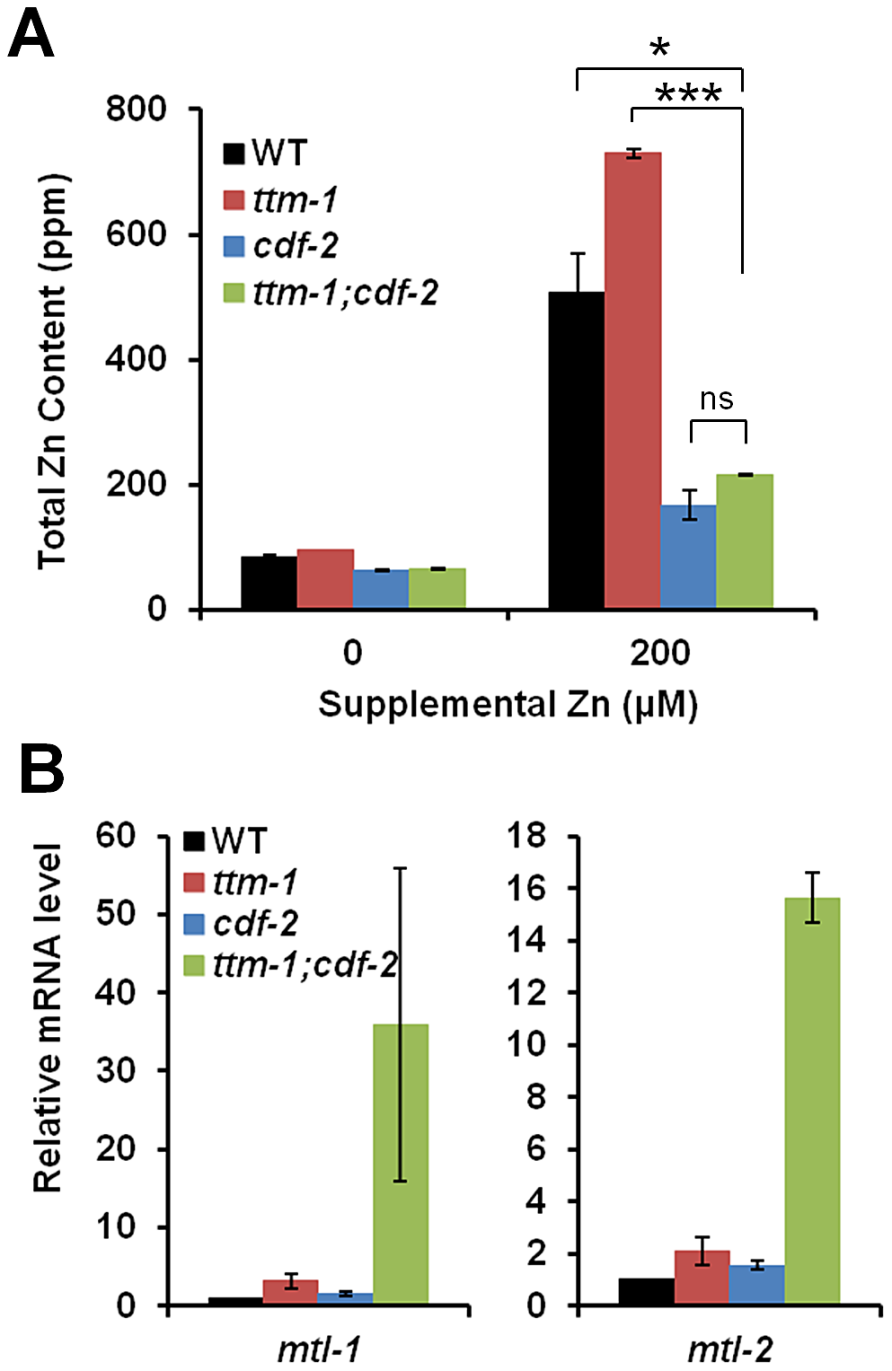
Relationships between *ttm-1* and *cdf-2*. (A) Total zinc content of wild-type, *ttm-1(ok3503)*, *cdf-2(tm788)*, and *ttm-1(ok3503);cdf-2(tm788)* animals analyzed by ICP-MS. Bars indicate average ± SEM of two independent experiments (ns, p>0.05, *p<0.05, ***p<0.005). The wild type and *ttm-1(ok3503)* data are the same as Figure 6A. (B) mRNA levels of *mtl-1* (left) or *mtl-2* (right) in animals cultured with no supplemental zinc determined by quantitative RT-PCR. The bars indicate the average ± SEM of three independent experiments. The mRNA level in WT animals was set equal to 1.0, and other samples were relative to that sample. *ttm-1;cdf-2* double mutant animals displayed elevated levels of *mtl-1* and *mtl-2* mRNA compared to WT and single mutant animals in all three experiments, indicating this is a reproducible result. However, the combined data did not reach statistical significance at the level of p<0.05 because the values of the fold changes varied between the experiments.

To further characterize the relationship between *ttm-1* and *cdf-2*, we analyzed *ttm-1;cdf-2* double mutant animals for total zinc content using ICP-MS. In the presence of 0 µM and 200 µM supplemental zinc, *ttm-1;cdf-2* mutant animals displayed lower total zinc content than wild-type animals, similar to *cdf-2* mutant animals (Figure 8A). These results indicate that although the *ttm-1* mutation causes a defect in zinc excretion, the *cdf-2* mutation prevents this excess zinc from being stored in gut granules, resulting in an overall decrease in organismal zinc. Thus, CDF-2 is required for the hyperaccumulation of zinc in *ttm-1* mutant animals, and TTM-1 isoforms and CDF-2 act antagonistically with respect to total organismal zinc content.

To indirectly measure the level of cytoplamic zinc in intestinal cells, we determined mRNA levels of *mtl-1* and *mtl-2* using RT-PCR. In the absence of supplemental zinc, *ttm-1;cdf-2* double mutant animals displayed strikingly elevated levels of *mtl-1* and *mtl-2* expression compared to wild-type animals, approximately 35-fold and 15-fold increases, respectively (Figure 8B). With 200 µM supplemental zinc, the expression of *mtl-1* and *mtl-2* in *ttm-1;cdf-2* double mutant animals was further elevated (Figure S4A and S4B). These results indicate that both *ttm-1* and *cdf-2* function to inhibit *mtl-1/2* expression, presumably by reducing the level of cytoplasmic zinc in intestinal cells. Furthermore, the synergistic effect of combining the two mutations suggests that *ttm-1* and *cdf-2* function redundantly to decrease cytoplasmic levels of zinc. Together, the findings that total zinc content decreased whereas *mtl-1/2* expression dramatically increased suggests that *mtl-1/2* gene expression responds to zinc levels in the cytoplasm but does not respond to zinc levels in the storage gut granules. Thus, impaired zinc storage in *ttm-1;cdf-2* double mutant animals causes a reduction in gut granule zinc content and an overall decrease in total zinc content. By contrast, the combination of impaired zinc storage and excretion causes an increase in the levels of cytoplasmic zinc that induces expression of *mtl-1/2*. These highly elevated levels of *mtl-1* and *mtl-2* expression were not observed in *ttm-1;cdf-1* or *ttm-1;sur-7* mutant animals (Figure S4C–S4F), indicating the specificity of the genetic interaction between *ttm-1* and *cdf-2*. These results indicate that zinc homeostasis is severely impaired in *ttm-1;cdf-2* mutant animals and suggest that TTM-1 isoforms and CDF-2 are key regulators of zinc homeostasis that act in a coordinated manner to control the levels of cytoplasmic zinc.

## Discussion

### *ttm-1* is a complex locus that encodes two CDF protein isoforms

By exhaustively searching the *C. elegans* genome for CDF proteins, we identified 14 predicted family members. *C. elegans* contains one or more proteins that are highly similar to all ten human CDF proteins, indicating that mechanisms of zinc metabolism are conserved in nematodes and mammals. We previously demonstrated that *C. elegans* CDF-2 plays a critical role in detoxification by transporting zinc into lysosome-related organelles in intestinal cells [30]. Here we focused on the *cdf* gene *ttm-1*, because TTM-1 isoforms are highly related to CDF-2, and these *C. elegans* proteins are similar to human ZnT2, ZnT3, ZnT4 and ZnT8.

The analysis of *ttm-1* demonstrated that the locus generates two transcripts, *ttm-1a* and *ttm-1b*, that use alternative transcription start sites. The *ttm-1a* and *ttm-1b* promoters appear to function independently based on the analysis of reporter constructs in transgenic animals. Both *ttm-1a* and *ttm-1b* were expressed in intestinal cells, but *ttm-1a* was also expressed in hypodermal cells whereas *ttm-1b* was also expressed in neurons and seam cells. Although endogenous TTM-1 proteins were not visualized, these expression patterns are likely to reflect the endogenous expression patterns, since constructs containing these promoters expressing TTM-1 isoforms fused to GFP were capable of rescuing the zinc hypersensitivity phenotype of *ttm-1* mutant animals. A second difference between the promoters was revealed by the response to high zinc conditions. *ttm-1b* mRNA levels were increased in response to high levels of dietary zinc, whereas *ttm-1a* mRNA levels were not changed. The *ttm-1b* promoter was sufficient to confer zinc-inducibility when fused to the reporter GFP, indicating that regulation occurs at the level of transcriptional initiation. The *cis*-acting DNA elements and *trans*-acting proteins that mediate transcriptional induction have not been defined.

The *ttm-1a* and *ttm-1b* transcripts encode proteins that have unique N-termini and share a common C-terminus. Both TTM-1A and TTM-1B contain six predicted membrane spanning domains and a histidine-rich motif, suggesting both protein isoforms are functional zinc transporters. The TTM-1A and TTM-1B isoforms displayed distinct subcellular localizations. In intestinal cells, TTM-1B was localized to the apical surface of the plasma membrane, whereas TTM-1A was localized to intracellular vesicles. TTM-1A-containing vesicles did not colocalize with mitochondria or gut granules, which are lysosome-related organelles. Although the identity of the TTM-1A-containing vesicles was not further characterized, the homology of TTM-1A with CDF-2 and ZnT2 raises the possibility that TTM-1A may be localized to a distinct lysosomal compartment. Because the two TTM-1 isoforms have unique N-terminal sequences, we infer that the N-terminal regions contain amino acid motifs that target the isoforms to distinct subcellular localizations. Furthermore, the TTM-1B isoform displayed a different subcellular localization in different cell types; TTM-1B was localized to intracellular vesicles in neurons and seam cells. Thus, TTM-1B may respond to cell type-specific mechanisms for subcellular localization and play distinct roles in zinc metabolism in different cell types. These results illustrate how a single zinc transporter locus can make complicated contributions to zinc metabolism by generating two protein isoforms that are expressed in overlapping and unique tissues, have different subcellular localizations and respond differently to dietary zinc.

### *ttm-1* mediates zinc excretion by intestinal cells

**Figure 9:**
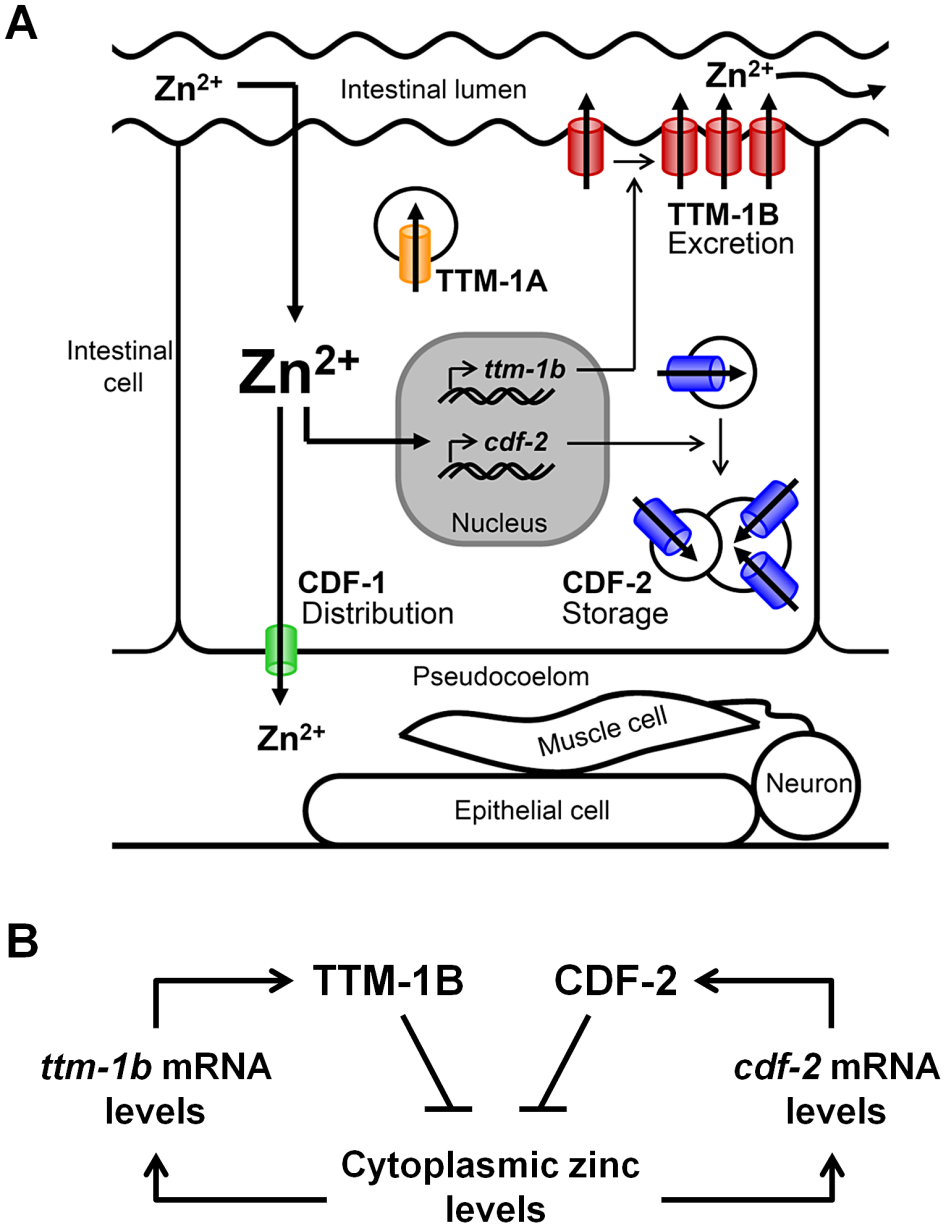
Network of CDF zinc transporters in intestinal cells. (A) Dietary zinc moves from the intestinal lumen to the cytoplasm of intestinal cells by an undefined mechanism. High levels of cytoplasmic zinc increase transcription of *ttm-1b* and *cdf-2*. CDF-1 (green) functions in distribution; it is localized to the basolateral surface of the plasma membrane of intestinal cells and transports cytoplasmic zinc into the pseudocoelum for use by cells such as epithelia, muscles and neurons. TTM-1B (red) functions in excretion; it is localized to the apical surface of the plasma membrane of intestinal cells and transports cytoplasmic zinc into the intestinal lumen. CDF-2 (blue) functions in storage: it is localized to the membrane of gut granules, lysosome-related organelles that acquire a bilobed morphology in response to high zinc, and it transports zinc into the lumen of these organelles [30]. TTM-1A (yellow) is localized to vesicles and may also promote zinc excretion and/or sequestration. (B) A parallel negative feedback circuit promotes zinc homeostasis in intestinal cells. TTM-1B and CDF-2 proteins reduce the level of cytoplasmic zinc and promote zinc detoxification by distinct mechanisms of zinc excretion and zinc sequestration, respectively. *ttm-1b* and *cdf-2* transcripts are both induced by high levels of cytoplasmic zinc. Mutations that reduce the activity of *ttm-1* or *cdf-2* cause induction of the remaining protein.

To determine the function of *ttm-1* in zinc metabolism, we analyzed a deletion mutation that lacks the coding region for highly conserved domains and is likely to severely reduce the activity of both the TTM-1A and TTM-1B isoforms. Zinc levels in *ttm-1* mutant animals were evaluated using three independent methods; the measurement of total zinc content by ICP-MS, the analysis of *mtl-1/2* transcript levels by RT-PCR, and the visualization of labile zinc using a zinc-responsive fluorescent dye, Zinpyr-1. *ttm-1* mutant animals displayed significantly elevated total zinc content, demonstrating excess zinc is present somewhere in the animal. To define the anatomical location(s) of the excess zinc, we showed that *ttm-1* mutant animals displayed elevated *mtl-1* and *mtl-2* mRNA levels. Because these genes are transcribed specifically in intestinal cells and are likely to respond to the level of cytoplasmic zinc, these results suggest that *ttm-1* mutant animals have elevated levels of cytoplasmic zinc in intestinal cells. Because TTM-1B is localized to the apical surface of the plasma membrane in intestinal cells, these results suggest that TTM-1B contributes to zinc homeostasis by promoting zinc excretion into the intestinal lumen (Figure 9A).

Using the newly developed technique of Zinpyr-1 staining, we demonstrated that there is a pool of labile zinc in the pseudocoelom. We hypothesized that the source of pseudocoelomic zinc is excretion from intestinal cells mediated by CDF-1, the homolog of vertebrate ZnT1 [26]. Consistent with this hypothesis, we showed that CDF-1 was localized specifically on the basolateral surface of the plasma membrane of intestinal cells. Importantly, *cdf-1* mutant animals displayed reduced Zinpyr-1 staining in the pseudocoelom. We previously reported that total zinc content is elevated in *cdf-1* mutant animals [28], suggesting that *cdf-1* mutant animals accumulate zinc in intestinal cells as a result of defective zinc transport into the pseudocoelom. *ttm-1* mutant animals displayed higher Zinpyr-1 fluorescence in the pseudocoelom than wild-type animals, indicating that *ttm-1* is necessary to limit the level of zinc in the pseudocoelom. The high Zinpyr-1 fluorescence in *ttm-1* mutant animals was decreased in *ttm-1;cdf-1* double mutant animals. Based on these results, we propose that *ttm-1* functions in intestinal cells to promote zinc excretion into the intestinal lumen. In *ttm-1* mutant animals, zinc accumulates in the cytoplasm of intestinal cells, and some of this excess zinc is transported into the pseudocoelom by CDF-1 (Figure 9A).

TTM-1A and TTM-1B in intestinal cells were localized to intracellular vesicles and the apical surface of the plasma membrane, respectively. The subcellular localization of TTM-1B indicates a direct role in excretion by transport of zinc across the apical surface of the plasma membrane. The subcellular localization of TTM-1A is consistent with an indirect role in excretion, if TTM-1A-containing vesicles fuse with the apical surface of the plasma membrane, or a role in sequestration. To analyze the function of each isoform separately, we generated transgenic animals that expressed only TTM-1A or TTM-1B in the background of *ttm-1(ok3503)*. Both isoforms displayed rescue activity, indicating that both isoforms can be functionally important. Two additional experiments highlight the importance of TTM-1B. First, TTM-1B but not TTM-1A was induced by high levels of dietary zinc specifically in intestinal cells, suggesting TTM-1B plays a more prominent role during zinc excess. Second, the intestine-specific expression of TTM-1B alone was sufficient to rescue the zinc hypersensitivity phenotype of *ttm-1;cdf-2* mutant animals. These results suggest that the direct transport of zinc across the apical plasma membrane of intestinal cells by TTM-1B plays an important and physiological role.

The yeast *Saccharomyces cerevisiae* does not appear to excrete zinc, since CDF proteins are not localized to the plasma membrane [41]. To detoxify excess zinc, yeast cells sequester zinc in an intracellular vesicle, the vacuole. In mammals, pancreatic enzymes and bile that contain zinc are a source of zinc excretion in feces [18]. However, the primary purpose of pancreatic enzymes and bile is nutrient absorption, not zinc homeostasis, and the role of enterocytes in zinc excretion has not been well defined. Our discovery of zinc excretion from the apical plasma membrane of *C. elegans* intestinal cells mediated by a conserved CDF protein raises the possibility that this mechanism may be conserved in mammals.

Several mammalian CDF proteins are expressed in enterocytes, including ZnT1, ZnT2, ZnT4, ZnT5, ZnT6 and ZnT7 [19]. The protein sequence of ZnT2 is highly similar to TTM-1 isoforms, and ZnT2 has intriguing functional similarities. ZnT2 expression is induced by high dietary zinc, and two isoforms of ZnT2 result from alternative splicing; one isoform localizes to the plasma membrane, whereas and the other isoform localizes to intracellular endosome/lysosome-like vesicles [22], [42]. ZnT2 promotes zinc excretion from mammary epithelial cells [7]. Similarly, ZnT4 has been localized to the plasma membrane and intracellular vesicles, its subcellular localization changes in response high zinc conditions, and it functions in zinc secretion from mammary epithelial cells [19]. The ZnT5 gene encodes two isoforms, ZnT5A and ZnT5B, and each isoform exhibits a distinct subcellular localization; ZnT5A is localized to the Golgi complex, whereas ZnT5B is localized to the apical membrane of enterocytes in the small intestine [24], [43]. Functional studies of ZnT5B using cell lines and *Xenopus* oocytes suggest that ZnT5B may be a bidirectional zinc transporter that mediates both zinc influx and efflux [25]. These results suggest that ZnT2, ZnT4 and/or ZnT5B may function in the excretion of zinc from enterocytes into the intestinal lumen, but these functions have not been demonstrated *in vivo*.

### TTM-1B and CDF-2 act in a parallel negative feedback circuit to promote zinc homeostasis

To analyze how the activity of multiple zinc transporters is coordinated to promote homeostasis, we analyzed genetic interactions between *ttm-1* and previously characterized *cdf* genes. *ttm-1*;*cdf-2* double mutant animals displayed a dramatic induction of *mtl-1/2* mRNA levels, indicating that the level of cytoplasmic zinc is highly elevated in intestinal cells. Thus, the coordinated actions of TTM-1B in zinc excretion and CDF-2 in zinc storage maintain cytoplasmic levels of zinc in intestinal cells. Consistent with this model, *ttm-1;cdf-2* double mutant animals displayed extreme sensitivity to dietary zinc, indicating these genes function coordinately to promote zinc detoxification. Similar defects in zinc homeostasis were observed when the *ttm-1* mutation was combined with mutations in *glo* genes that cause defects in gut granule biogenesis and zinc storage. These results confirm that the genetic interaction of *ttm-1* with *cdf-2* reflects the role of CDF-2 in zinc storage in gut granules of intestinal cells (Figure 9A). The genetic interaction between *ttm-1* and *cdf-2* was specific, since reducing the activity of *ttm-1* did not strongly affect the zinc sensitivity of *cdf-1* and *sur-7* mutant animals.

Our results indicate that coordination between *ttm-1* and *cdf-2* occurs at two levels; one level is transcriptional control, and a second level is protein function. Here we demonstrated that reducing the activity of *ttm-1* resulted in increased expression of *cdf-2* mRNA. We previously showed that reducing the activity of *cdf-2* resulted in increased expression of *ttm-1* mRNA [30]. These results suggest that *ttm-1* mutant animals and *cdf-2* mutant animals have elevated cytoplasmic zinc in intestinal cells, which induces expression of zinc-regulated transcripts, including *ttm-1* and *cdf-2*. At the level of protein function, both CDF-2 and TTM-1B function to reduce the level of cytoplasmic zinc, thereby enhancing zinc detoxification and reducing expression of zinc-regulated transcripts. This is a negative feedback circuit because rising levels of cytoplasmic zinc induce CDF-2 and TTM-1B protein expression, which in turn reduce the levels of cytoplasmic zinc. This is a parallel circuit since CDF-2 and TTM-1B proteins reduce cytoplasmic zinc by independent mechanisms: storage in lysosome-related organelles and excretion from intestinal cells, respectively. Because *ttm-1b* and *cdf-2* are both zinc inducible, and both proteins promote low levels of cytoplasmic zinc, we propose that these genes constitute a parallel negative feedback circuit that maintains zinc homeostasis (Figure 9B).

Whereas *cdf-2* single mutant animals displayed measurable zinc hypersensitivity, *ttm-1* single mutant animals appeared to be similar to wild type in zinc sensitivity. One interpretation of these results is that zinc storage mediated by CDF-2 is the primary response to high zinc conditions and zinc excretion mediated by TTM-1B is the secondary response. The primacy of zinc storage relative to excretion may be a strategy for optimal handling of excess zinc. Zinc is an essential nutrient, and the availability of zinc may fluctuate in natural environments. When exposed to zinc abundance, it may be strategic for animals to first store zinc and later excrete excess zinc, thereby optimizing preparation for future exposure to zinc-deficient conditions. Therefore, the coordinated activity of CDF-2 mediated zinc storage and TTM-1B mediated zinc excretion may maintain zinc homeostasis and also optimize storage to promote survival in environments with fluctuating zinc levels.

## Materials and Methods

### General methods and strains

*C. elegans* strains were cultured at 20°C on nematode growth medium (NGM) with a lawn of *E. coli* OP50 for food [44]. The wild-type strain and parent of all mutant strains was Bristol N2. The following mutations were used: *pgp-2(kx48) I* [40], *glo-1(zu391) X* [39], *cdf-1(n2527) X* [26], *cdf-2(tm788) X* [28] and *sur-7(ku119) X* [31]. *ttm-1(ok3503) III* was generated by the *C. elegans* Gene Knockout Project at OMRF, which is part of the International *C. elegans* Gene Knockout Consortium. We backcrossed *ttm-1(ok3503)* five times with N2 before analysis. The molecular lesion of *ttm-1(ok3503)* was defined by determining the DNA sequence of the *ttm-1* locus that was PCR-amplified using the following primers: cccgccaaaaattattcaga and accgtaatgggacagacagc. Double mutant animals were generated by standard methods, and genotypes were confirmed by PCR or DNA sequencing.

### Identification of *C. elegans* CDF proteins and phylogenetic analysis

To identify *C. elegans* CDF proteins, we conducted an iterative sequence-based homology search using PSI-BLAST [35]. Briefly, we searched open reading frames (ORFs) of *C. elegans* with the CDF-1 protein sequence and identified proteins with an E-value of 10^−3^ or less. These protein sequences were used to search for additional *C. elegans* ORFs with high similarity. To analyze evolutionary conservation, we used *C. elegans* CDF proteins to search ORFs of *S. cerevisiae*, *A. thaliana* and humans using PSI-BLAST. The identified orthologs were analyzed by reciprocal PSI-BLAST; each was used to search *C. elegans* ORFs to determine the most similar proteins. Multiple sequence alignment of CDF proteins was carried out using ClustalW, and the resulting alignment was used to generate a phylogenetic tree using MEGA [45].

### 5′ rapid amplification of cDNA ends (5′ RACE)

RNA was isolated from synchronized wild-type animals at the adult stage using TRIzol (Invitrogen), and 5′RACE was performed using the 5′ RACE System for Rapid Amplification of cDNA Ends Version 2.0 according to the manufacturer's protocol (Invitrogen). Briefly, RNA was reverse-transcribed into cDNA using a gene specific primer (GSP1) which hybridized to exon 4 of *ttm-1*. cDNA was tailed with oligo(dC), PCR-amplified using Abridge Anchor Primer and another gene specific primer (GSP2) that is positioned approximately 30 bp upstream of GSP1, and fractionated by agarose gel electrophoresis. Two PCR products of different sizes were observed, and the DNA sequence of each was determined. GSP1 is gtaaccgaatgaaagacgct, and GSP2 is gagaattcaagacgtgcacaacgaatcg.

### Quantitative real-time PCR (RT–PCR)

Eggs were isolated from gravid adult hermaphrodites by bleaching, allowed to hatch in M9 buffer overnight, and the worms were cultured on NGM dishes for approximately 2.5 days. Synchronized animals at L4/young adult stage were washed and then cultured on noble agar minimum medium (NAMM) dishes supplemented with zinc sulfate (ZnSO_4_) and seeded with concentrated OP50. After 16–24 hr, animals were washed and collected for RNA isolation. RNA analysis was performed as previously described with modifications [28]. Briefly, RNA was isolated using TRIzol (Invitrogen) and treated with DNase I, and cDNA was synthesized using the High-Capacity cDNA Reverse Transcription kit according to the manufacturer's protocol (Applied Biosystems). PCR was performed using an Applied Biosystems 7900 Fast Real-Time PCR System thermocycler and SYBR Green PCR Master Mix (Applied Biosystems). Fold change was determined by comparing target gene expression with the reference gene expression (*ama-1* and *rps-23*) under the same conditions. The primers used for PCR were: *ama-1*, atcggagcagccaggaactt and gactgtatgatggtgaagctgg; *rps-23*, aaggctcacattggaactcg and aggctgcttagcttcgacac; *ttm-1a*, aacgttttcgacggaggagg and ctctctcgactctggcaacc; *ttm-1b*, catgggcactcacacacacac and ctcggcgacccttttgatatttc; *cdf-2*, atagcaatcggagagcaacg and tgtgacaattgcgagtgagc; *mtl-1*, ggcttgcaagtgtgactgc and cctcacagcagtacttctcac; *mtl-2*, ggtctgcaagtgtgactgc and gcagcagtattgctcacagc.

### Plasmid DNA construction

For the *ttm-1a* promoter fusion construct, the region between the *ttm-1a* start codon and the 3′ end of the adjacent upstream gene (∼1.2 kb) was PCR amplified using genomic DNA from wild-type animals. For the *ttm-1b* promoter, the region extending ∼6 kb upstream of the *ttm-1b* start codon was generated by PCR amplifying two fragments of DNA using genomic DNA from wild-type animals. We inserted these promoter regions, GFP coding sequence, and the *unc-54* 3′UTR into pBluescript SK+ (Stratagene) to generate pHR17 [*Pttm-1a::GFP*] and pHR6 [*Pttm-1b::GFP*]. For TTM-1 translational fusion constructs, *ttm-1a* cDNA was PCR amplified using EST clone yk1572h06 that was obtained from the National Institutes of Genetics in Japan. *ttm-1b* cDNA was generated by combining exon 3 and exon 4–5 fragments that were PCR amplified from EST clone yk1572h06 and wild-type genomic DNA, respectively. The appropriate cDNA (without the stop codon) was inserted into pHR17 and pHR6 to generate pHR4 [*Pttm-1a*::*TTM-1A::GFP*] and pHR7 [*Pttm-1b::TTM-1B::GFP*]. *ttm-1b* cDNA was also inserted into pSC7 [*Pcdf-2::GFP*] to generate pHR8 [*Pcdf-2::TTM-1B::GFP*]. For the *cdf-1* translational fusion construct, the genomic DNA fragment containing the *cdf-1* promoter and coding sequence were inserted with GFP and the *unc-54* 3′UTR into MM016 to generate pDP13 [*Pcdf-1::CDF-1::GFP;unc-119(+)*].

### Transgenic strain generation and microscopy

We generated transgenic animals containing extrachromosomal arrays by injecting hermaphrodites with pHR4, pHR6, pHR7, pHR8, or pHR17 and the coinjection marker pCJF104 [*Pmyo-3::mCherry*] and selecting F1 progeny expressing mCherry in body wall muscles. We generated transgenic animals containing integrated arrays by bombardment transformation with pDP13 into *unc-119(ed3)* animals [46]. We selected progeny that were non-Unc and segregated only non-Unc progeny.

For live fluorescence microscopy, animals were paralyzed in a drop of 10 mM levamisole in M9 buffer on 2% agarose pads on microscope slides. Immunostaining of transgenic animals was performed as described by Davis et al [28]. Briefly, animals were fixed in methanol and acetone, rehydrated, and stained with a rabbit anti-GFP antibody (Invitrogen) and an Alexa Fluor 488 goat anti-rabbit secondary antibody (Invitrogen). Fluorescence was visualized using a Zeiss Axioplan 2 microscope equipped with a Zeiss AxioCam MRm digital camera.

### Zinpyr-1 staining

Zinpyr-1 (USBiological, Z0530) was reconstituted in dimethylsulfoxide (DMSO) to generate a 5 mM stock solution and diluted in M9 buffer to a final concentration of 20 µM. Hermaphrodites at the L4/young adult stage were cultured for 12–16 h on NAMM dishes supplemented with ZnSO_4_ and seeded with concentrated OP50. Animals were washed, transferred into 20 µM Zinpyr-1 in M9, and incubated for 3–4 h in the dark. Animals were washed three times with M9, paralyzed in 10 mM levamisole in M9, mounted on 2% agarose pads on microscope slides, and visualized as described above. For comparison, images were captured using identical settings and exposure times. To quantify Zinpyr-1 staining, we categorized individual worms into four groups based on visual inspection according to the fluorescence intensity and pattern; Low was defined as no detectable or weak fluorescence, Medium was defined as low level fluorescence in a wide area, High was defined as moderate fluorescence in a wide area and strong fluorescence in a small area, and Highest was defined as strong fluorescence in a wide area of the body.

### Inductively coupled plasma-mass spectrometry (ICP-MS)

Metal content analysis was performed as previously described [30], with modifications. For sample preparation, large populations of animals were generated by culturing on multiple 100 mm NGM dishes. Animals were washed and cultured on multiple 100 mm NAMM dishes supplemented with ZnSO_4_ and seeded with concentrated OP50. After ∼24 h, animals were washed three times in magnesium-free (Mg-free) M9 containing 0.01% Tween-20, incubated in 1 mM serotonin in Mg-free M9 for 30 min to remove bacteria from the intestinal lumen, washed twice in Mg-free M9, transferred to preweighed tubes and frozen at −80°C. For ICP-MS, samples were freeze-dried, reweighed to obtain the dry pellet weight, and digested by incubation in a hot block digester with concentrated nitric acid and hydrogen peroxide solution. The solution was diluted with water, and internal standards were added to correct for matrix effects. Instrument calibration standards were prepared from multi-element stock solutions (High-Purity Standards) to generate a linear calibration curve, and samples were analyzed using a Perkin Elmer NexION ICP-MS. The zinc content was determined as a value in parts-per-million (ppm) by dividing zinc weight by dry worm pellet weight (µg/g).

### Zinc sensitivity assays

Zinc sensitivity assays were conducted as previously described [30]. Briefly, eggs were isolated from gravid adult hermaphrodites by treating with NaOH and bleach and allowed to hatch in M9 overnight. Synchronized L1 animals were then cultured on NAMM dishes supplemented with ZnSO_4_ and seeded with concentrated OP50. After ∼3 days, animals were washed, paralyzed with 10 mM sodium azide (NaN_3_) in M9, mounted on a 2% agarose pad on a microscope slide, and visualized as described above. The length of animals was measured using ImageJ software (NIH) by drawing a line from the nose to the tail tip.

## Supporting Information

Figure S1 5′ RACE of *ttm-1* isoforms. The sequence of 50 nucleotides of the 5′ end of *ttm-1a* (top) and *ttm-1b* (bottom) transcripts identified by the method of 5′RACE. The SL1 trans-spliced leader (red) is present in both transcripts, followed by 3 or 4 nucleotides before the predicted protein coding sequence begins with the ATG start codon (box).
(TIF)

Figure S2 Definition of the molecular lesion in the *ttm-1(ok3503)* allele. The genomic DNA sequence of the *ttm-1* locus starting in exon 4 (shown in uppercase) and extending to intron 4 (shown in lowercase). The 877 bp region deleted in the *ttm-1(ok3503)* allele is shown in blue.
(TIF)

Figure S3 Intestine-specific expression of TTM-1B::GFP. Fluorescence microscope images of transgenic *ttm-1(ok3503);cdf-2(tm788)* animal expressing TTM-1B::GFP under the control of the *cdf-2* promoter [*Pcdf-2::TTM-1B::GFP;ttm-1;cdf-2*]. Animals at late L4 or young adult stage were cultured with 0 µM or 100 µM supplemental zinc. Images display the entire animal from the head (left) to the tail (right). Images were captured with the identical settings and exposure times. The scale bar represents 50 µm.
(TIF)

Figure S4 RT-PCR analysis of *mtl-1* and *mtl-2* mRNAs. (A–F) *mtl-1* and *mtl-2* mRNA levels were analyzed by RT-PCR in the animals cultured with the indicated concentrations of supplemental zinc. Genotypes were wild-type, *ttm-1(ok3503)*, *cdf-2(tm788)*, *ttm-1(ok3503);cdf-2(tm788)*, *cdf-1(n2527)*, *ttm-1(ok3503);cdf-1(n2527)*, *sur-7(ku119)*, and *ttm-1(ok3503);sur-7(ku119)*. The bars indicate the average ± SEM of three independent experiments. Wild type cultured in 0 µM supplemental zinc was set equal to 1.0 for each panel, and the other samples were relative to that sample. The data at 0 µM supplemental zinc in panels (A) and (B) are the same as those shown in Figure 8B but illustrated on a different scale. The *ttm-1;cdf-1* mutant animals with no supplemental zinc did not display a consistent induction of *mtl-1* and *mtl-2*.
(TIF)
