# Supplementary figures and images for: ttm-1 Encodes CDF Transporters That Excrete Zinc from Intestinal Cells of C. elegans and Act in a Parallel Negative Feedback Circuit That Promotes Homeostasis
Source: PLoS Genet. 2013 May 23;9(5):e1003522. doi: 10.1371/journal.pgen.1003522 (PMC3662639; doi:10.1371/journal.pgen.1003522)

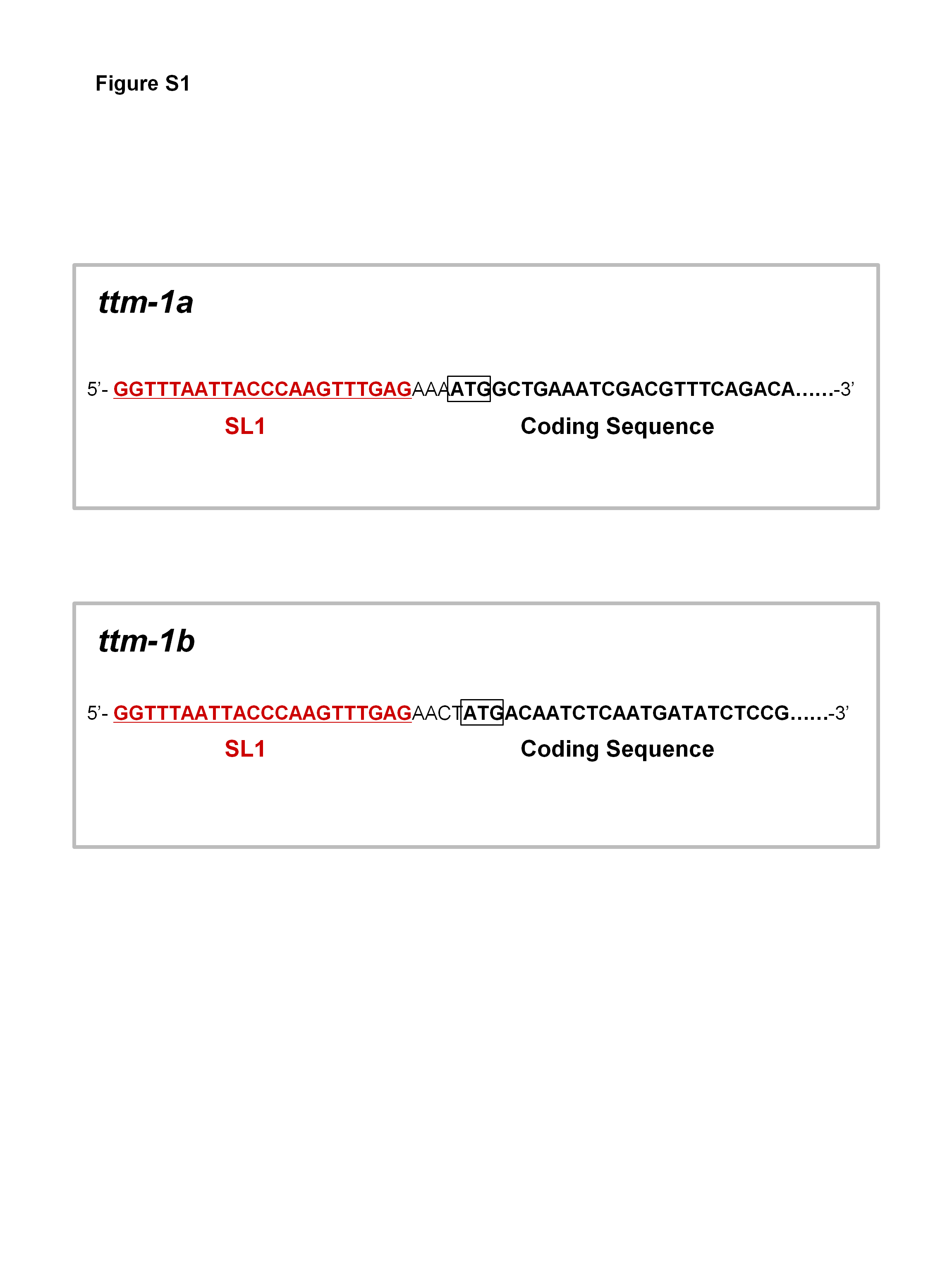

Supplement: Figure S1 — 5′ RACE of ttm-1 isoforms. The sequence of 50 nucleotides of the 5′ end of ttm-1a (top) and ttm-1b (bottom) transcripts identified by the method of 5′RACE. The SL1 trans-spliced leader (red) is present in both transcripts, followed by 3 or 4 nucleotides before the predicted protein coding sequence begins with the ATG start codon (box). (TIF) [file pgen.1003522.s001.tif]

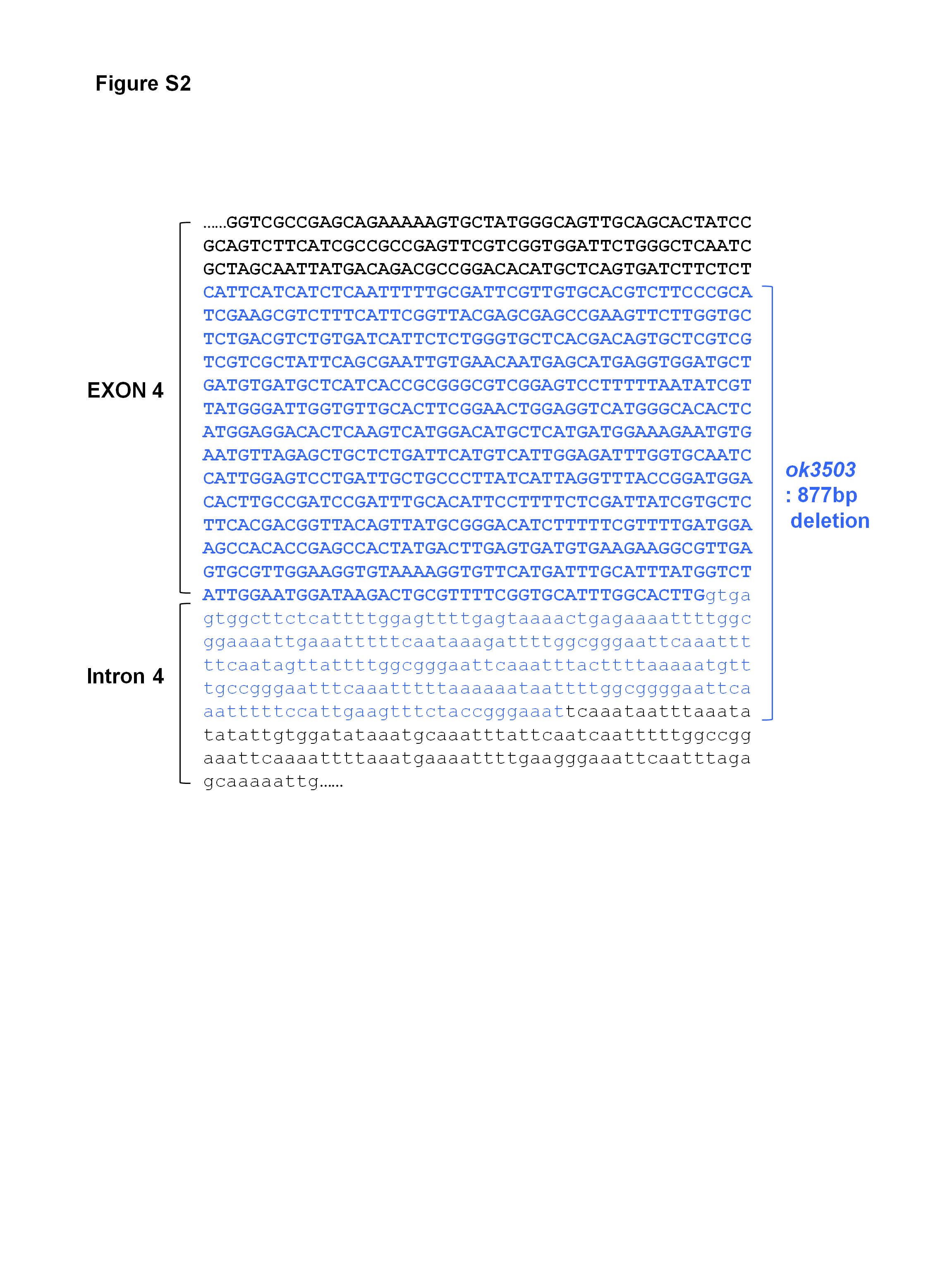

Supplement: Figure S2 — Definition of the molecular lesion in the ttm-1(ok3503) allele. The genomic DNA sequence of the ttm-1 locus starting in exon 4 (shown in uppercase) and extending to intron 4 (shown in lowercase). The 877 bp region deleted in the ttm-1(ok3503) allele is shown in blue. (TIF) [file pgen.1003522.s002.tif]

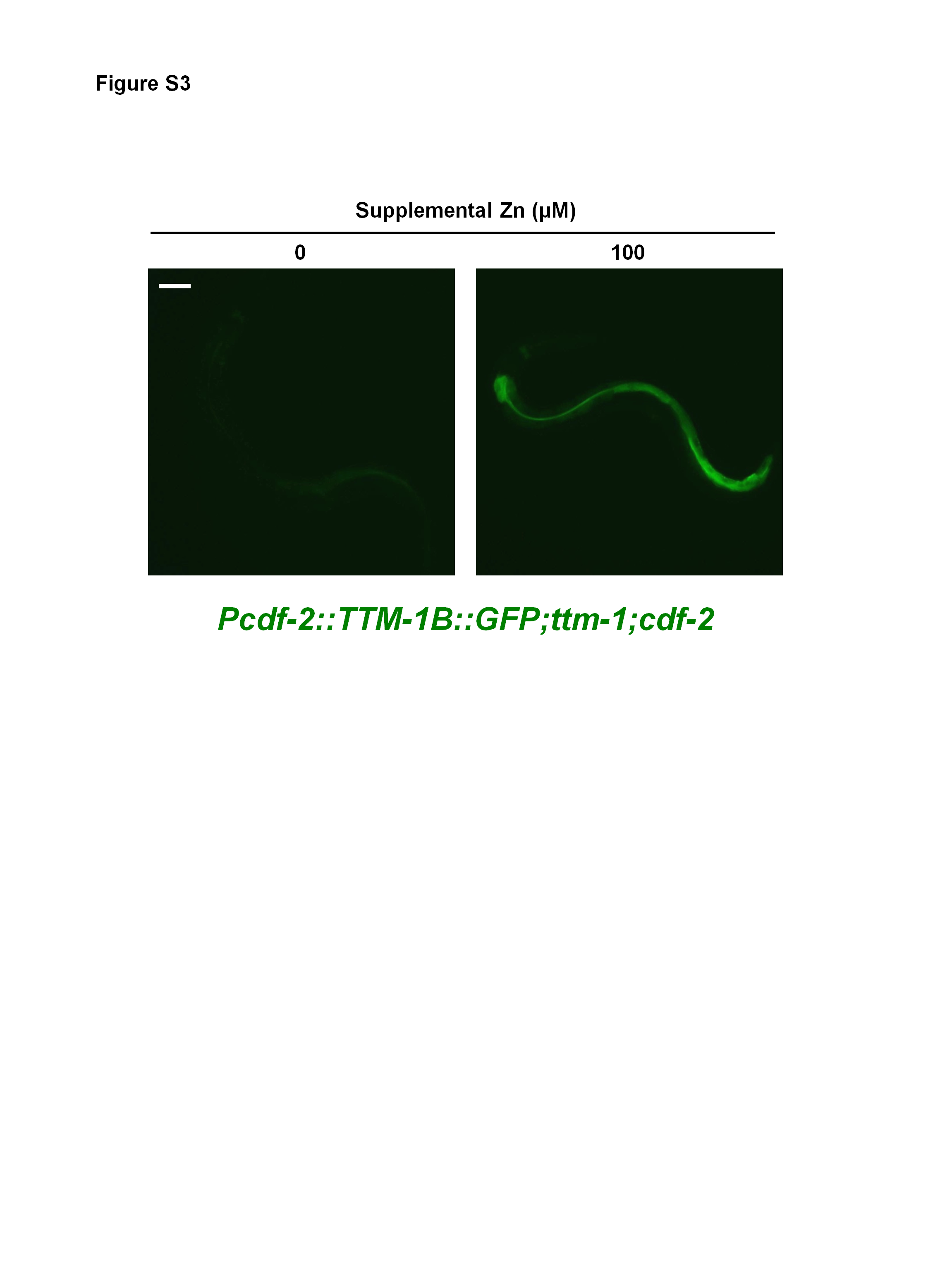

Supplement: Figure S3 — Intestine-specific expression of TTM-1B::GFP. Fluorescence microscope images of transgenic ttm-1(ok3503);cdf-2(tm788) animal expressing TTM-1B::GFP under the control of the cdf-2 promoter [Pcdf-2::TTM-1B::GFP;ttm-1;cdf-2]. Animals at late L4 or young adult stage were cultured with 0 µM or 100 µM supplemental zinc. Images display the entire animal from the head (left) to the tail (right). Images were captured with the identical settings and exposure times. The scale bar represents 50 µm. (TIF) [file pgen.1003522.s003.tif]

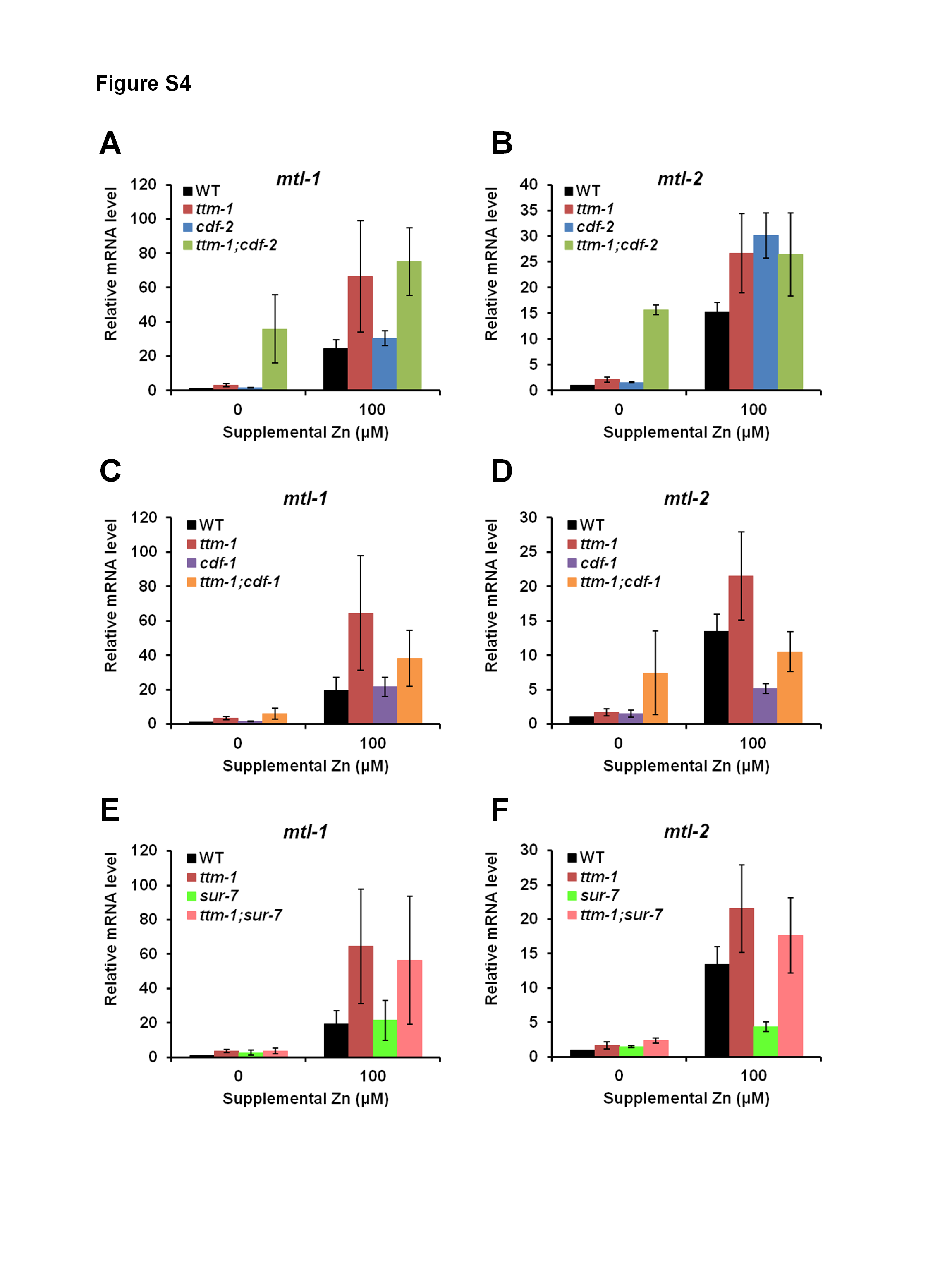

Supplement: Figure S4 — RT-PCR analysis of mtl-1 and mtl-2 mRNAs. (A–F) mtl-1 and mtl-2 mRNA levels were analyzed by RT-PCR in the animals cultured with the indicated concentrations of supplemental zinc. Genotypes were wild-type, ttm-1(ok3503), cdf-2(tm788), ttm-1(ok3503);cdf-2(tm788), cdf-1(n2527), ttm-1(ok3503);cdf-1(n2527), sur-7(ku119), and ttm-1(ok3503);sur-7(ku119). The bars indicate the average ± SEM of three independent experiments. Wild type cultured in 0 µM supplemental zinc was set equal to 1.0 for each panel, and the other samples were relative to that sample. The data at 0 µM supplemental zinc in panels (A) and (B) are the same as those shown in Figure 8B but illustrated on a different scale. The ttm-1;cdf-1 mutant animals with no supplemental zinc did not display a consistent induction of mtl-1 and mtl-2. (TIF) [file pgen.1003522.s004.tif]
